# Supplementary material for: The Autophagy Receptor TAX1BP1 (T6BP) improves antigen presentation by MHC‐II molecules
Source: EMBO Rep. 2022 Oct 10;23(12):e55470. doi: 10.15252/embr.202255470 (PMC9724678; doi:10.15252/embr.202255470)
Supplement: Supplementary file 1 — Appendix [file EMBR-23-e55470-s001.pdf]

Appendix

Table of content:

Appendix Figure-S1 (related to Figure-1) page-2

Appendix Figure-S2 (related to Figure-4) page-3

Appendix Figure-S3 (related to Figure-5) page-4

Appendix Figure-S4 (related to Figure-6) page-5

Appendix Table S1 (related to EV4) page-6

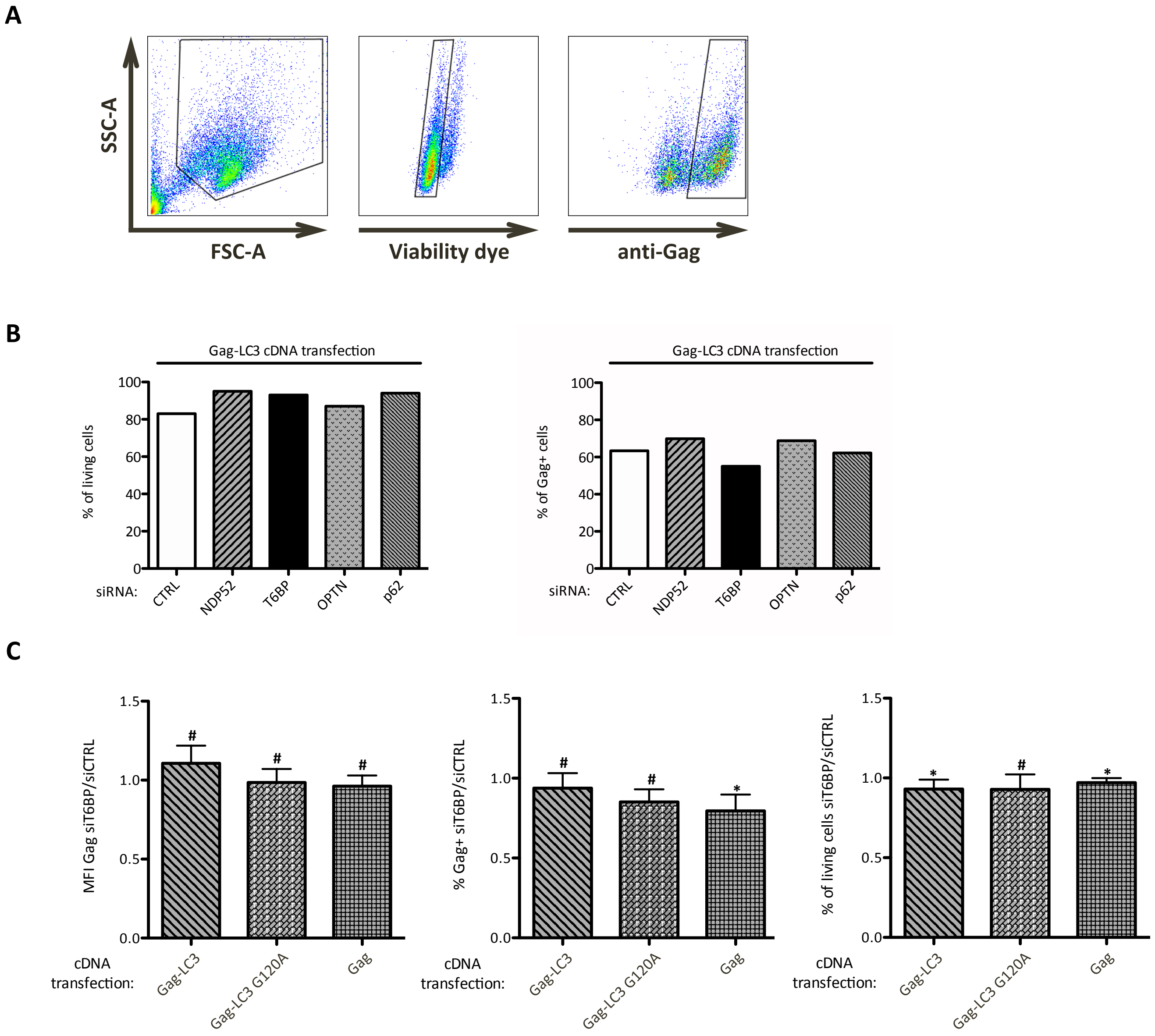

Appendix Figure-S1 (related to Figure-1): The transfection of siRNA targeting autophagy receptors influenced neither the cell viability nor the antigen transfection efficiencies. (A) and (B) Analysis of cell viability and transfection efficiency using flow cytometry. Results of one representative experiment out of three are shown. **(A)** Gating strategy: left panel: FSC-A: forward-scatter; SSC-A: side-scatter; middle panel: staining of living cells using a viability dye; right panel: staining of Gag^{+} cells using anti-Gag antibody intracellular staining. **(B)** Percentage of living and Gag^{+} cells in the different siRNA transfection conditions, left and right panel respectively, analyzed 48h post-treatment, prior co-culture with Gag-specific T cells. **(C)** Results of at least three independent experiments are normalized to control conditions and presented as mean (+/- SD). Left panel, ratio of mean fluorescent intensities (MFI) of Gag stainings in Gag^{+} cells. Middle panel, ratio of the mean percentage of Gag^{+} cells. Right panel, ratio of the mean percentage of living cells. CTRL: control. Mann-Whitney's tests; *p<0.05; #p>0.05.

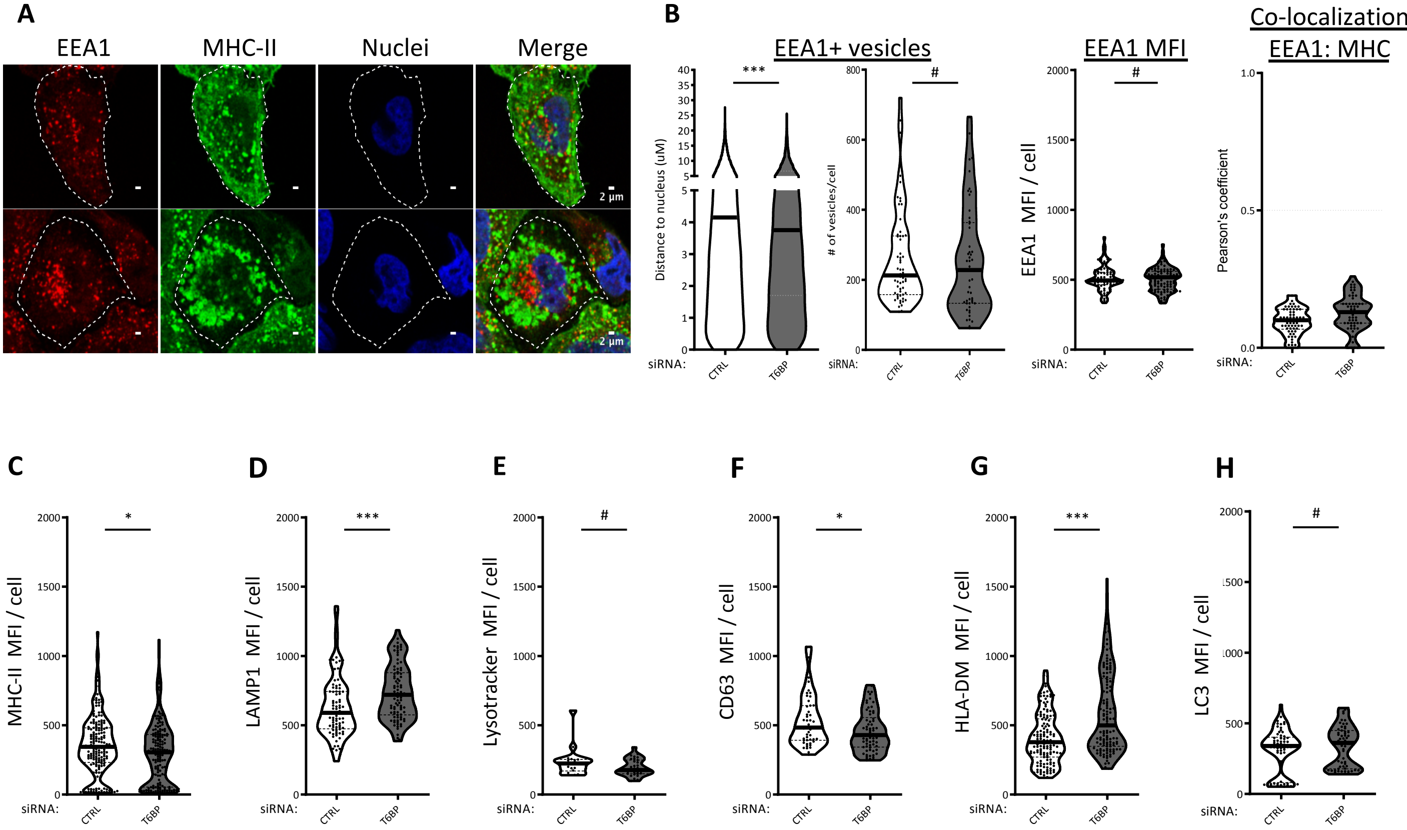

Appendix Figure S2 (related to Figure-4): T6BP silencing slightly influences EEA1+ vesicle cellular localization (A) EEA1 expression was assessed using confocal microscopy. HeLa-CIITA cells were transfected with siCTRL and siT6BP. 48h post-treatment, EEA1 were detected using specific antibody and a fluorescent secondary antibody. Nuclei were stained using DAPI. **(B)** Quantitative analysis using in-house ImageJ script displaying distance of each EEA1^{+} vesicles to the nucleus, number of EEA1^{+} vesicles per cell and EEA1 total MFI per cell. At least 16 000 vesicles from 90 cells corresponding to 3 independent experiments were analyzed. **(B, Right panel) EEA1 and MHC-II stainings do not colocalize.** Quantification of the potential colocalization between MHC-II^{+} and EEA1^{+} dots using Pearson's coefficient where the dotted lines (at 0.5) indicate the limit under which no significant co-localization is measured. **(C-H) Effect of siT6BP silencing on the expression levels of the various vesicular markers.** As in A, HeLa-CIITA cells were transfected with siCTRL and siT6BP. 48h post-treatment, cells were fixed and labeled and the MFI of the indicated markers were analyzed. **(C)** MHC-II, at least 170 cells corresponding to 5 independent experiments were analyzed. **(D)** LAMP-1, at least 85 cells corresponding to 3 independent experiments were analyzed. **(E)** Lysotracker, at least 30 cells corresponding to 2 independent experiments were analyzed. **(F)** CD63, at least 60 cells corresponding to 2 independent experiments were analyzed. **(G)** HLA-DM, at least 140 cells corresponding to 3 independent experiments were analyzed. **(H)** LC3, at least 60 cells corresponding to 2 independent experiments were analyzed. In graphs representing the number of vesicles or the MFI per cell, each dot displayed corresponds to a single cell. Scale bars, 2µm. CTRL: control. Mann-Whitney's tests; *: $p<0.05$; **: $p<0.002$; ***: $p<0.0003$; #: $p>0.05$.

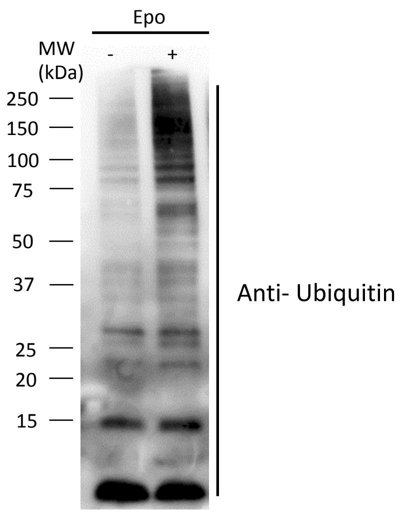

Appendix Figure-S3 (related to Figure-5D): Quality control of the proteasome inihibitor Epoxomicin (Epo) treatment. Hela-CIITA cells were treated with Epoxomicin (+) or mock treated (-) for 16h and total ubiquitin levels assessed using Western Blot. The membrane was blotted using an anti-ubiquitin antibody. MW: molecular weight markers.

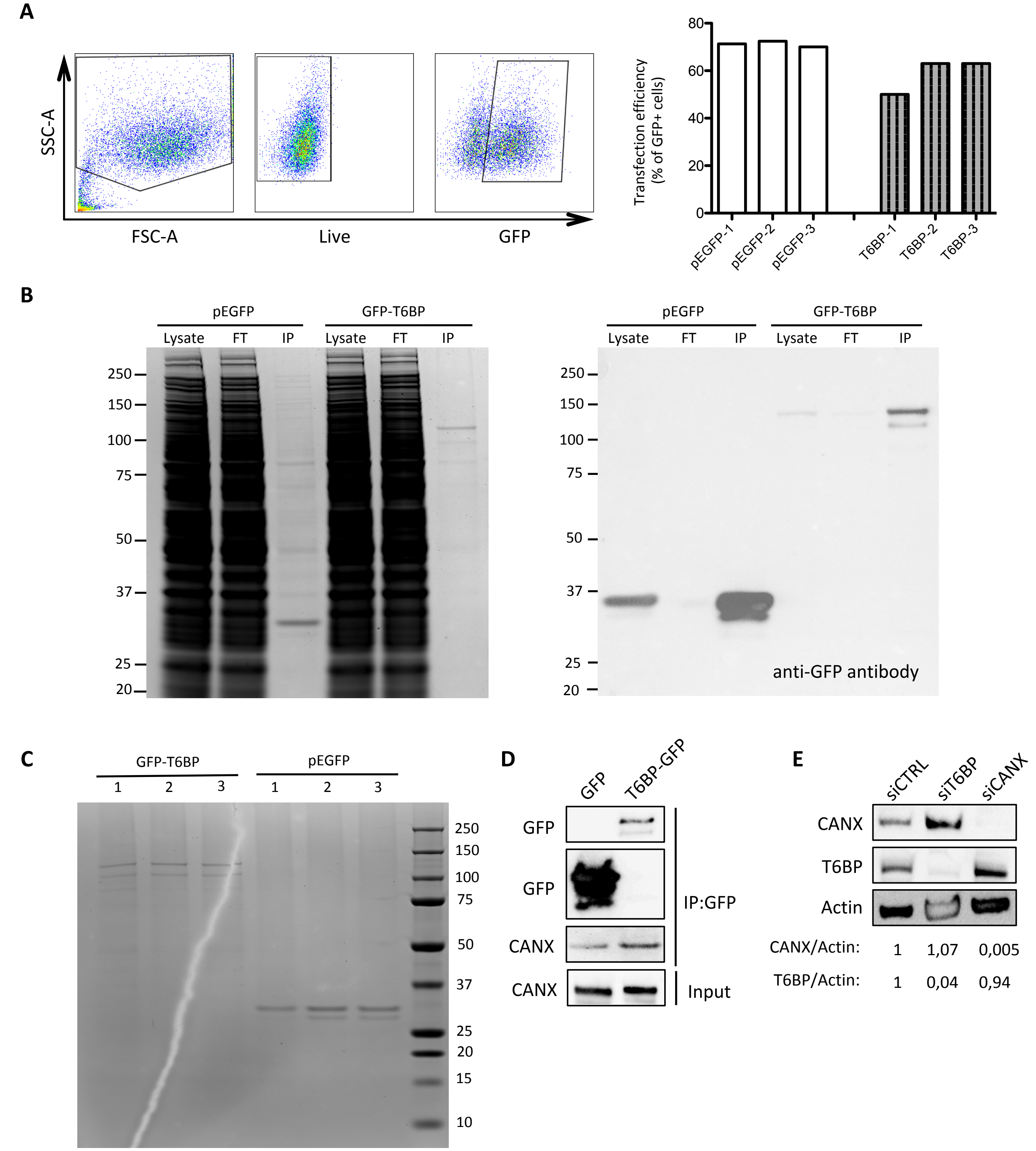

Appendix Fig S4 (related to Figure-6): Quality controls of T6BP interactome definition. (A) Left panels, gating strategy for the analysis of GFP+ cells after transfection of cDNA encoding wild-type T6BP fused to GFP using flow cytometry. FSC: forward-scatter; SSC: side-scatter. Right panels, side to side comparison of the percentage of GFP+ HeLa-CIITA cells transfected with plasmid encoding GFP-T6BP or GFP-only (pEGFP) in the three biological replicates. **(B)** Coomassie blue staining (left panel) and Western Blot analysis (right panel) of the indicated fraction of protein samples from GFP-T6BP and GFP expressing cells. Results from one of the three biological replicates are shown. Briefly, cells were lysed and submitted to IP using anti-GFP camel antibodies (GFP-Trap from Chromotek). Right panel, GFP and GFP-T6BP were revealed using anti-GFP antibody. FT = Flow through. IP = Immunoprecipitation. **(C)** Coomassie blue staining of immunoprecipitated proteins (1/10 of the sample volume) used for LC-MS/MS analysis (9/10 of the sample volume) of the three biological replicates for both GFP-T6BP and GFP. Due to a mishandling, a crack was unfortunately introduced to the gel when scanning **(D)** GFP nanobody immunoprecipitates from HeLa-CIITA cells transfected with GFP and T6BP-GFP. 48h post-transfection, IP (IP:GFP) and the total lysate (input) samples were analyzed by Western blot with the indicated antibodies. Input and FT of GFP or T6BP-GFP IP are presented in panel B. **(E)** Silencing of T6BP expression does not influence calnexin (CANX) expression levels. HeLa-CIITA cells transfected with the indicated siRNAs and samples analyzed, 48h post transfection, by Western blot with the indicated antibodies. The ratio of CANX or T6BP expression to actin was quantified using Image J and set at 1 [illegible]

Appendix Table S1 (related to EV4)

Primers and Probes	Sequence
CD74_S	gAATgCCACCAAgTATggCAA
CD74_R	gggggTCAgCATTCTggA
CD74_P TM	6FAM-CAggTgCATCACATggTCCTCTgT--BBQ
TAX1BP1_L	ggAgTCTTTCCACTggATTCAC
TAX1BP1_R	ggCCACATTTTgAAAgATgACA
TAX1BP1_P	6FAM- CCATTgCAgACTTCCAACTTTgCC--BBQ
ACTIN F	AgCCTCgCCTTTgCCgA
ACTIN R	CTggTgCCTggggCg
ACTIN TM	6FAM-CCgCCgCCCgTCCACACCCgCC--BBQ

Reverse transcription quantitative Polymerase Chain Reaction (RT-qPCR) primers: The primers and probes used for quantitation of CD74, T6BP and actin were designed by Olfert Landt and purchased from TIB MolBiol. Sequences are listed in the table below. The RT-qPCRs were performed in a Light Cycler 1.5 instrument in capillaries using a final volume of 20 µl. The reactions were performed using 300 nM specific sense primer, 300 nM specific antisense primer, 200 nM specific TaqMan probe (TM) and the LightCycler® Multiplex RNAVirus Master mix (ROCHE). The programs were: reverse-transcription 55°C for 10min, initial denaturation 95°C for 5min followed by 45 cycles of amplification. For CD74 and T6BP cycles were: 95°C for 5s, 60°C for 15s + fluorescence measurement and 72°C for 5s. For actin cycles were: 95°C for 20s, 67°C for 30s + fluorescence measurement and 72°C for 5s.
