# Supplementary material for: The Autophagy Receptor TAX1BP1 (T6BP) improves antigen presentation by MHC‐II molecules
Source: EMBO Rep. 2022 Oct 10;23(12):e55470. doi: 10.15252/embr.202255470 (PMC9724678; doi:10.15252/embr.202255470)
Supplement: Supplementary file 2 — Expanded View Figures PDF [file EMBR-23-e55470-s003.pdf]

Expanded View Figures

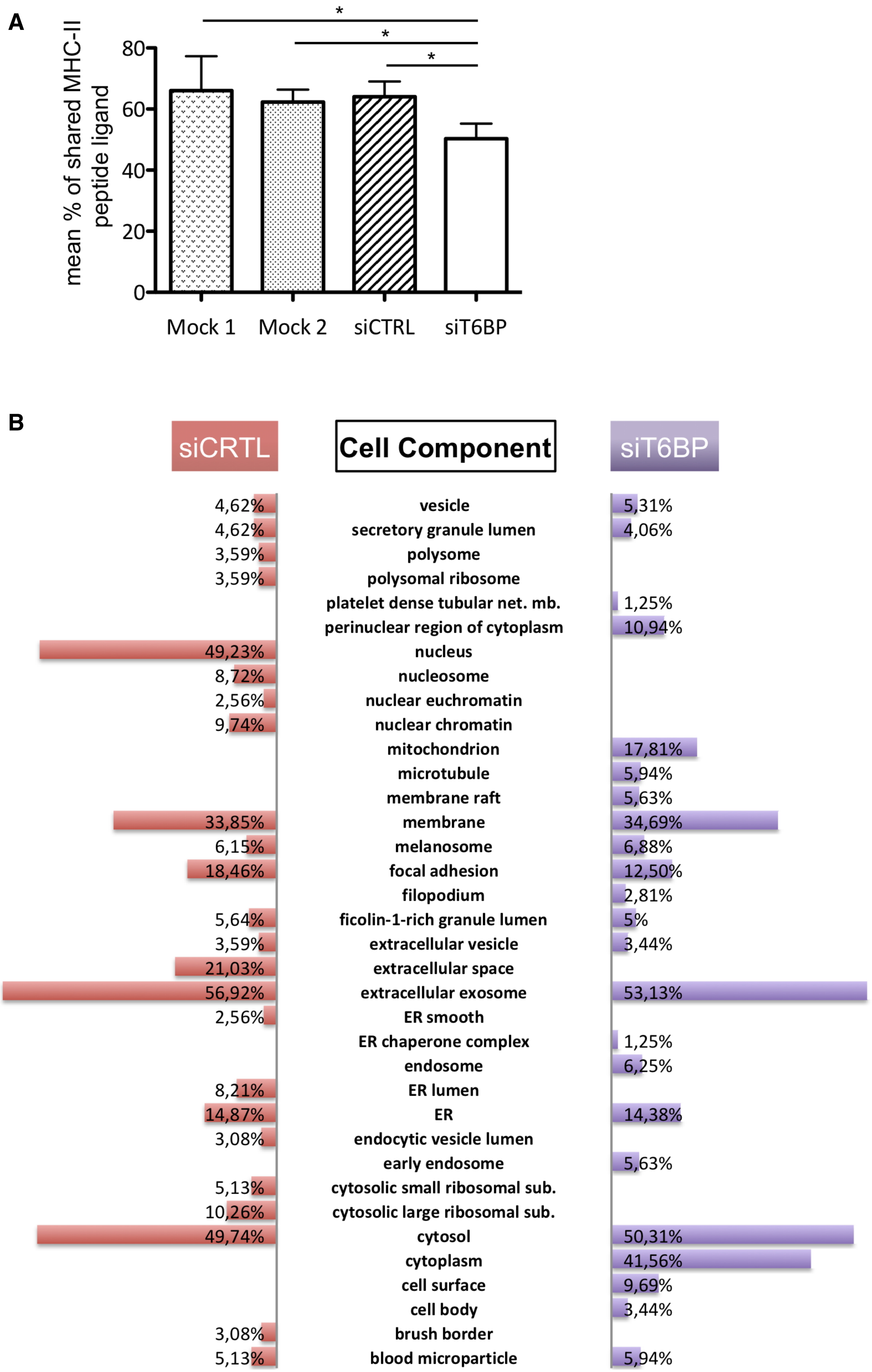

Figure EV1.

© 2022 The Authors

© 2022 The Authors

Figure EV1. (Related to Fig 3): T6BP silencing affects the repertoire of peptides presented by MHC-II molecules and has a modest influence on the source of MHC-II ligands.

A For each sample the % of MHC-II peptide ligand shared with the 3 other experimental conditions was determined and the mean % of shared MHC-II peptide ligand calculated and plotted (± SD). Comparing the mean % of shared peptides between mock treat cells (Mock1 or Mock2) and the cells transfected with the control (siCTRL) siRNA, no significant differences were observed. By contrast, the mean % of shared MHC-II peptide ligand was significantly different between siT6BP-treated cells and Mock1-, Mock2-, and siCTRL-treated cells. The statistical significance was calculated using a Kruskal–Wallis test followed by a Dunn's test (*$P < 0.05$).

B Cell component enrichment analysis of peptide sources. As in Fig 3, HeLa-CIITA cells were transfected with siCTRL and siT6BP siRNA, lysed, submitted to MHC-II immunoprecipitation using TÜ39 antibody, and the peptide ligands sequenced using mass spectrometry (LC–MS/MS). The diversity of protein sources was analyzed according to cell component enrichment using Funrich software. Only canonical pathways statistically enriched ($P < 0.05$) for each condition (siCTRL and siT6BP) are shown. The P-value for pathway enrichment was obtained using the right-tailed Fisher's exact test.

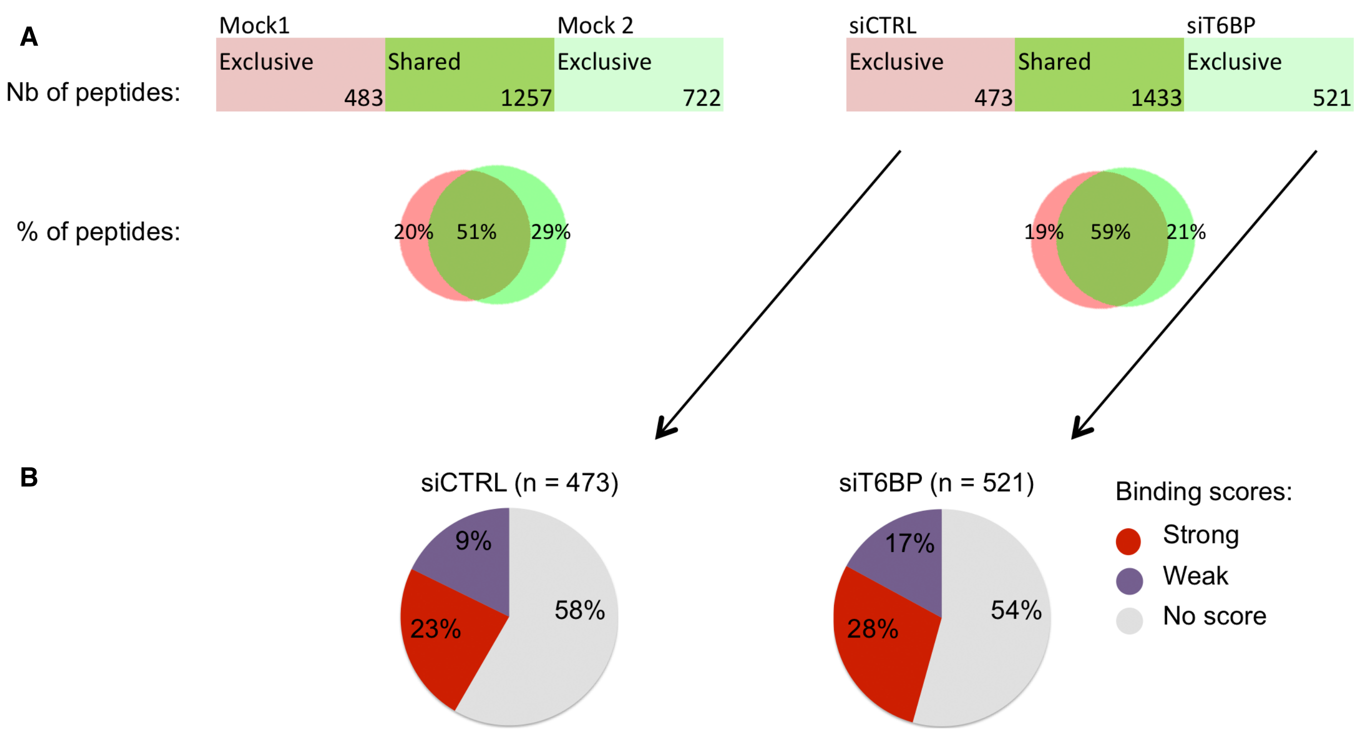

Figure EV2. (Related to Fig 3): T6BP silencing does not influence the immunopeptidome of MHC-I molecules.

A Left panel, mock-treated HeLa-CIITA cells were split and cultured for 48 h (giving rise to Mock1 and Mock2), then cells were lysed, MHC-I molecules were immunoprecipitated using W632 antibody and the peptide ligands sequenced using mass spectrometry (LC–MS/MS). Right panel, HeLa-CIITA cells were transfected with siCTRL and siT6BP siRNA and were treated as in the left panel. The number and the percentage among sequenced peptides (Venn diagrams) of exclusive or shared peptides for each condition are presented.

B Relative binding affinities, presented as pie charts, of exclusive peptides identified in siCTRL (left) and siT6BP (right) condition (number of peptides are indicated in brackets). NetMHCpan 4.0 algorithm was used to predict the relative affinities to HLA-A*6802 and -B*15093 molecules expressed by HeLa-CIITA cells. The relative affinities to the HLA-C*1203 molecule also expressed by HeLa-CIITA cells were not combined in this figure because many peptides binding to HLA-C*1203 also bind to HLA-A*6802. The results are presented as stated from NetMHCpan 4.0 analysis as Strong (for strong binders), Weak (for weak binders), and No score (for epitopes with which a binding score cannot be determined).

Data information: One representative experiment is shown out of two biological replicates. For each experiment, 5 technical replicates per sample were run on the LC–MS/MS. Nb—number; %—percentage.

© 2022 The Authors

Figure EV3. (Related to Fig 4). T6BP silencing leads to autophagosome accumulation.

A LC3 and T6BP expressions were assessed using confocal microscopy. HeLa-CIITA cells were transfected with siCTRL or siT6BP. 48 h post-treatment, LC3 and T6BP were detected using anti-LC3 and anti-T6BP antibodies, respectively. Scale bars: 2 μm.

B Quantitative analysis using in-house ImageJ script displaying the number of $LC3^+$ vesicles per cell and co-localization using Pearson's coefficient of T6BP and LC3 staining (right panel). 30 cells from two biological replicates were analyzed.

C, D As in A and B with MHC-II molecule and LC3 staining. A number of cells >40 from two biological replicates were analyzed. Scale bars: 2 μm.

E siRNA-treated cells were also analyzed using electron microscopy. Top panels and bottom panels, images from siCTRL- and siT6BP-treated cells, respectively, from 6 representative cells. Two biological replicates were performed and at least 40 cells for each treatment were analyzed. The white arrows indicate the autophagosomes. Scale bars: 1 μm.

F As in (A) with HLA-DM and T6BP staining. Scale bars: 4 μm.

G The localization of HLA-DM^+ vesicles and the number of vesicles/cells were quantified as in Fig 4. At least 10,000 vesicles in at least 40 cells were analyzed.

Data information: In graphs representing the number of vesicles/cell, each dot corresponds to a single cell. Within the violin plots, continuous and dotted lines correspond to medians and quartiles, respectively. CTRL: control; nb: number. Mann–Whitney's test; $*P < 0.05$; $***P < 0.0001$; ns >0.05. For Pearson's coefficient, the dotted lines (at 0.5) indicate the limit under which no significant co-localization is measured.

© 2022 The Authors

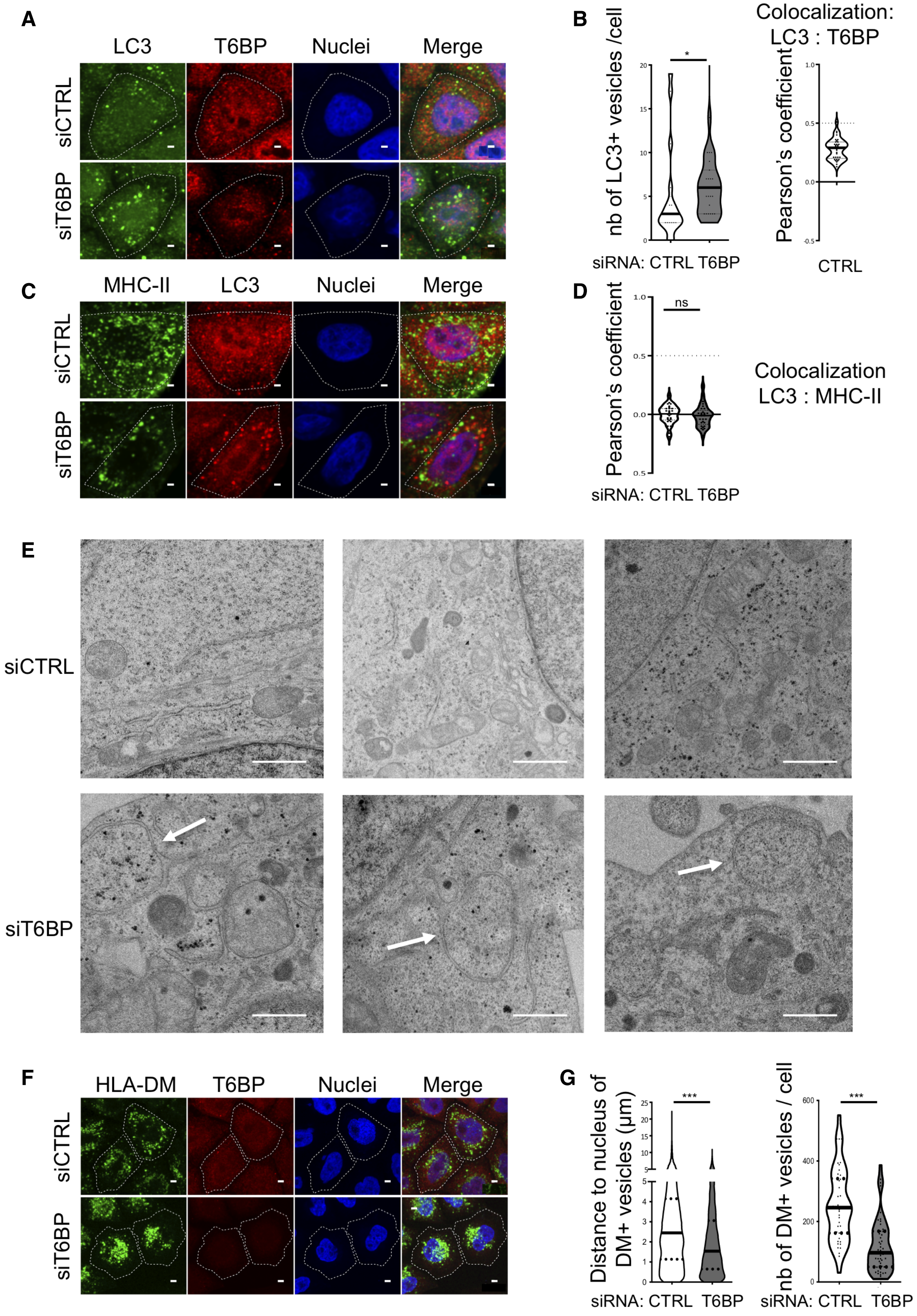
A
LC3
T6BP
Nuclei
Merge
siCTRL
siT6BP
B
nb of LC3+ vesicles /cell
*
siRNA: CTRL T6BP
Colocalization:
LC3 : T6BP
Pearson's coefficient
CTRL
C
MHC-II
LC3
Nuclei
Merge
siCTRL
siT6BP
D
Pearson's coefficient
ns
siRNA: CTRL T6BP
Colocalization
LC3 : MHC-II
E
siCTRL
siT6BP
F
HLA-DM
T6BP
Nuclei
Merge
siCTRL
siT6BP
G
Distance to nucleus of DM+ vesicles (μm)
***
siRNA: CTRL T6BP
nb of DM+ vesicles / cell
***
siRNA: CTRL T6BP

Figure EV3.

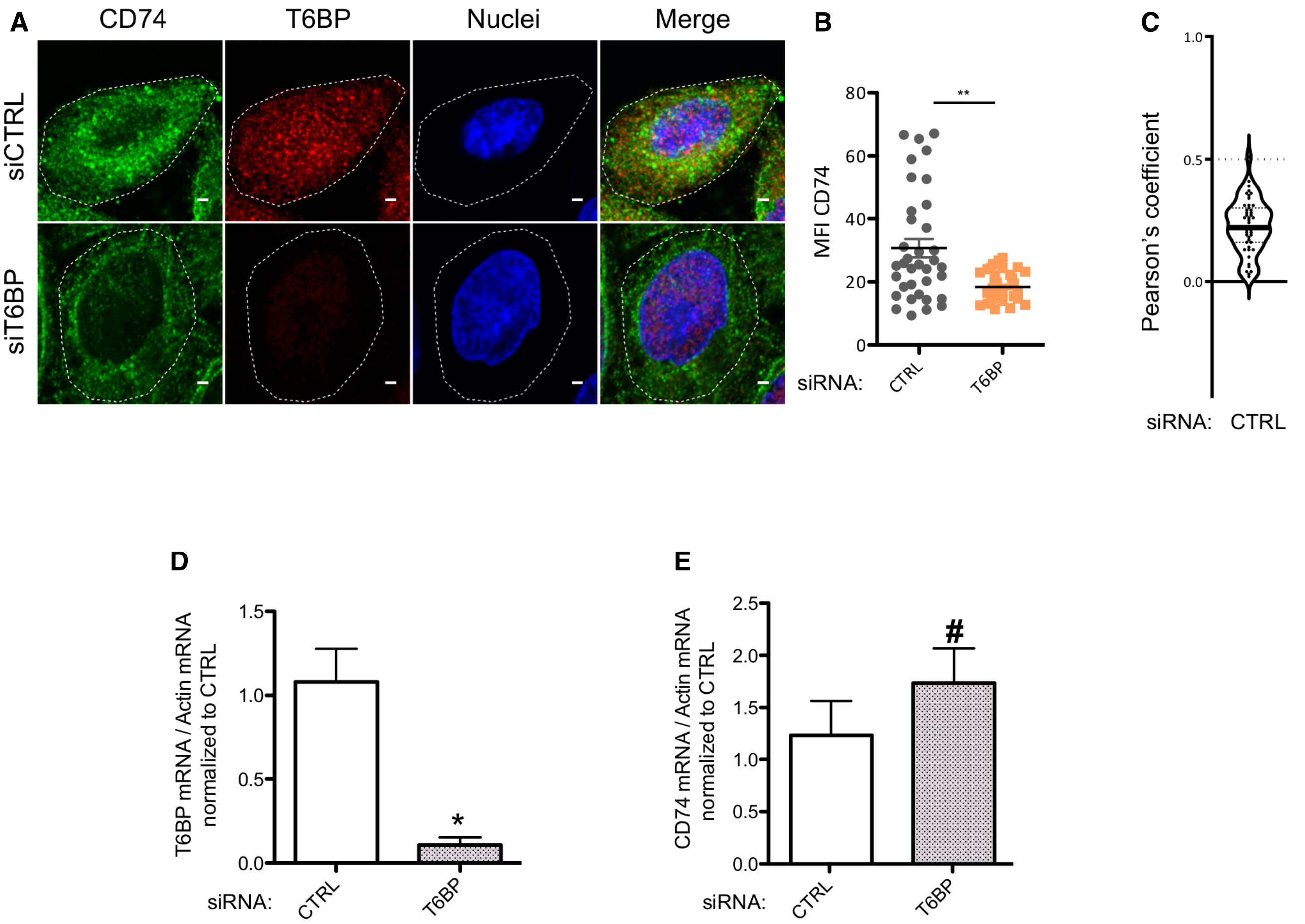

Figure EV4. (Related to Fig 5): T6BP silencing affects CD74 expression levels as assessed by confocal microscopy but does not affect CD74 mRNA levels.

A CD74 expression assessed using confocal microscopy in HeLa-CIITA cells, 48 h post-treatment with the indicated siRNA. Top panels siCTRL and bottom panels siT6BP. Scale bars: 2 µm.

B Quantitative analysis using ImageJ of CD74 mean fluorescent intensity (MFI). The data are representative of at least 3 biological replicates. Each dot displayed corresponds to a single cell. At least 75 cells were analyzed. The continuous lines represent the means (± SD). Mann–Whitney's test; $^{**}P < 0.002$.

C Co-localization of CD74 and T6BP assessed, in the control condition, using Pearson's coefficient. Number of cells = 47. Within the violin plots, continuous and dotted lines correspond to medians and quartiles, respectively. The dotted lines at 0.5 indicate the limit under which no significant co-localization is measured.

D, E (D) T6BP and (E) CD74 mRNA levels were assessed using RT–qPCR. HeLa-CIITA cells were transfected with siCTRL and siT6BP. 48 h post-treatment, relative T6BP (D) and CD74 (E) mRNA expression levels were analyzed by RT–qPCR using actin as reference gene. Results are presented as the mean ratios of T6BP (D) and CD74 mRNA (E) levels to actin mRNA levels (± SD) from four biological replicates. CTRL—control. Mann–Whitney's test: $^{*}P < 0.05$; $^{\#}P > 0.05$.

 © 2022 The Authors
