# Supplementary figures and images for: The Autophagy Receptor TAX1BP1 (T6BP) improves antigen presentation by MHC‐II molecules
Source: EMBO Rep. 2022 Oct 10;23(12):e55470. doi: 10.15252/embr.202255470 (PMC9724678; doi:10.15252/embr.202255470)

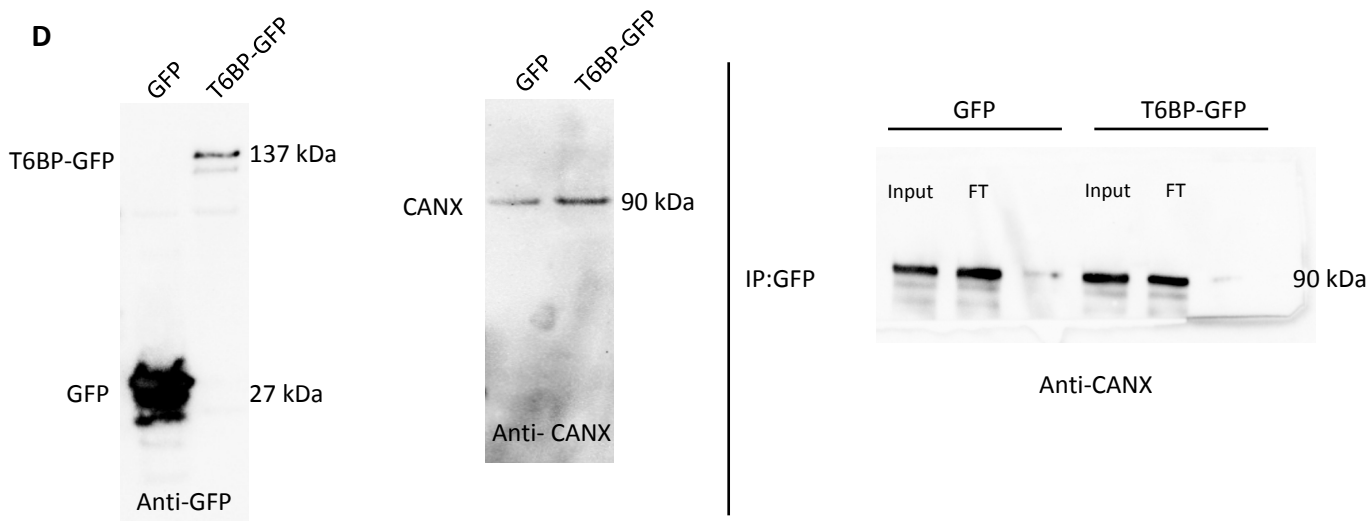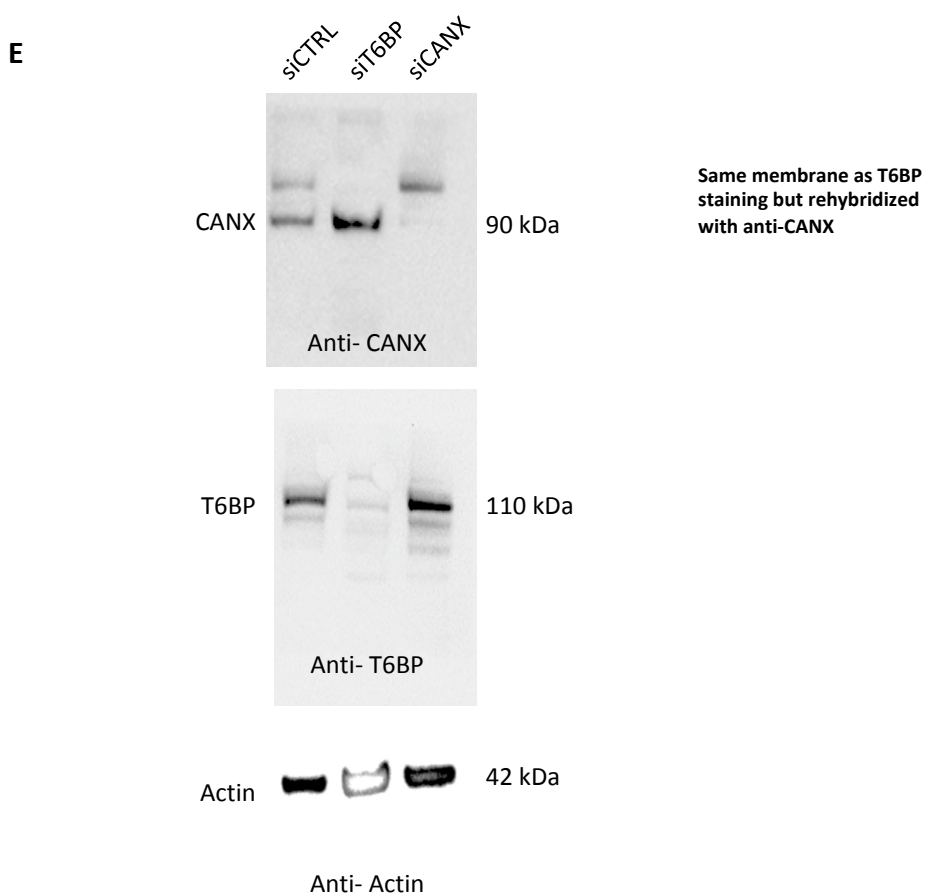

WB blot data for Appendix Figure S3

Supplement: Supplementary file 4 — Source Data for Appendix [file EMBR-23-e55470-s002.zip › EMBR_2273_Source_Data/Source_Data/EMBOR-2022-55470V2-WB_data_for_Appendix_Figure-S4-sd.pdf]

C

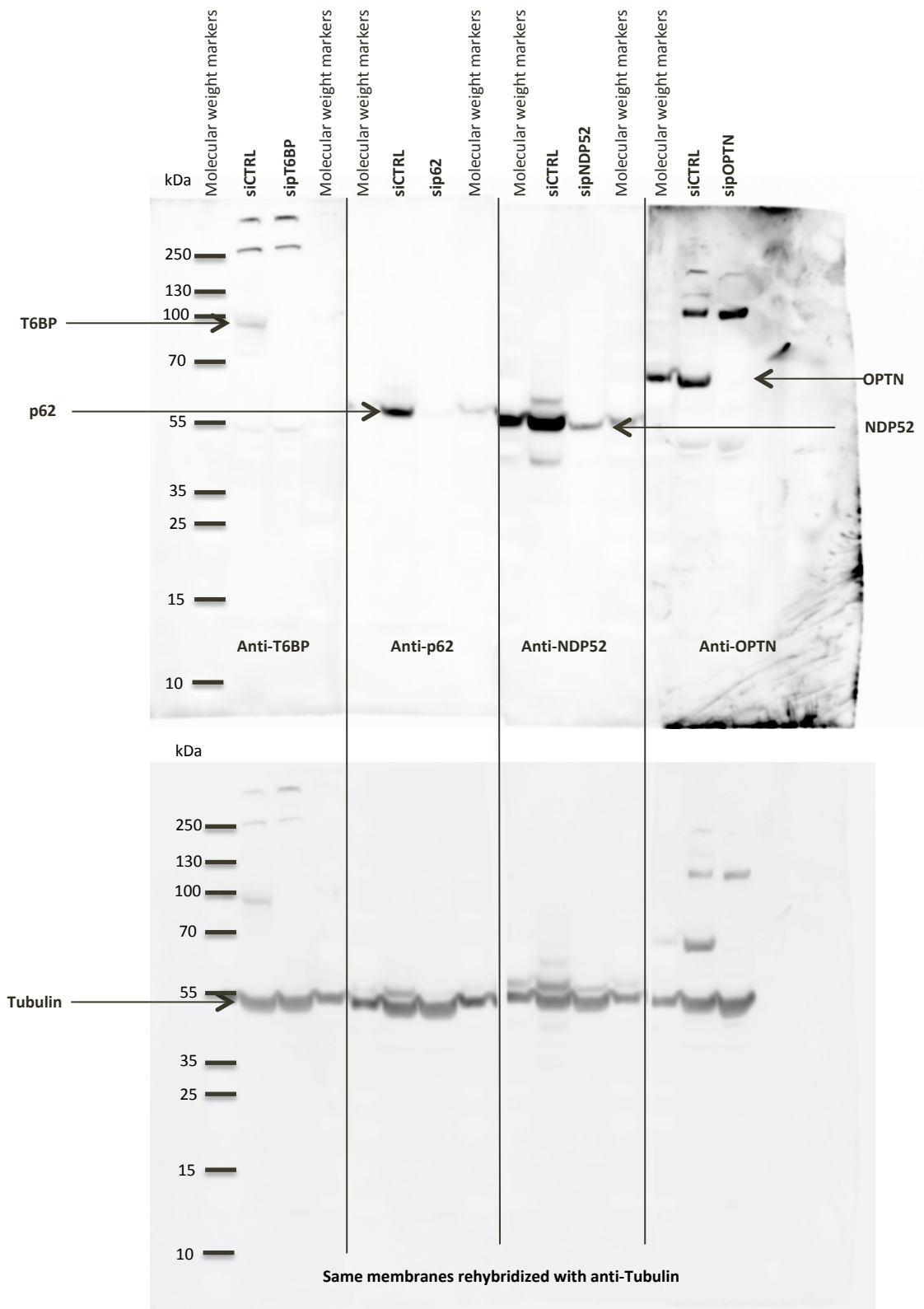

WB blot data for Figure 1C

Supplement: Supplementary file 5 — Source Data for Figure 1 [file EMBR-23-e55470-s007.pdf]

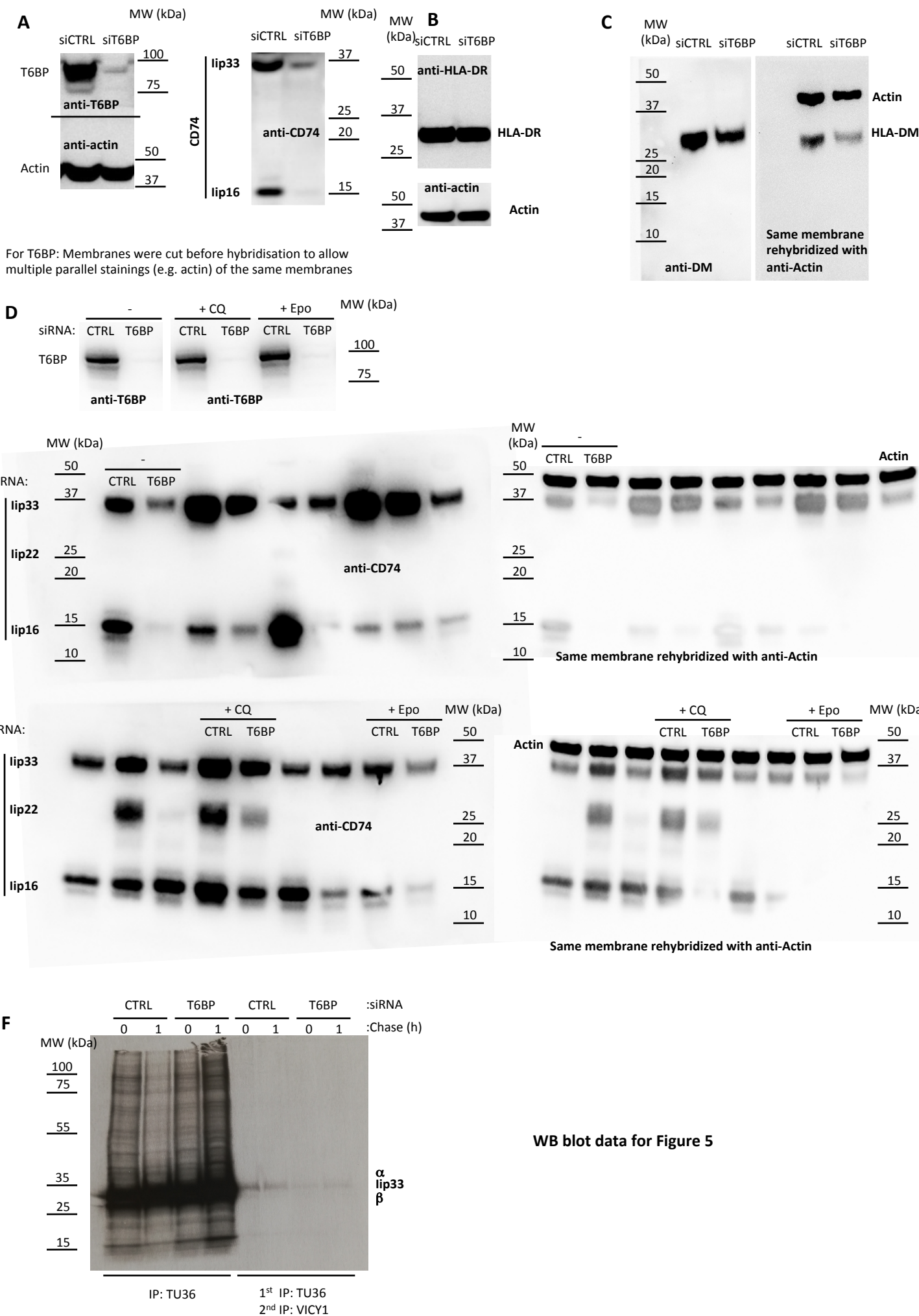

Supplement: Supplementary file 6 — Source Data for Figure 5 [file EMBR-23-e55470-s008.pdf]

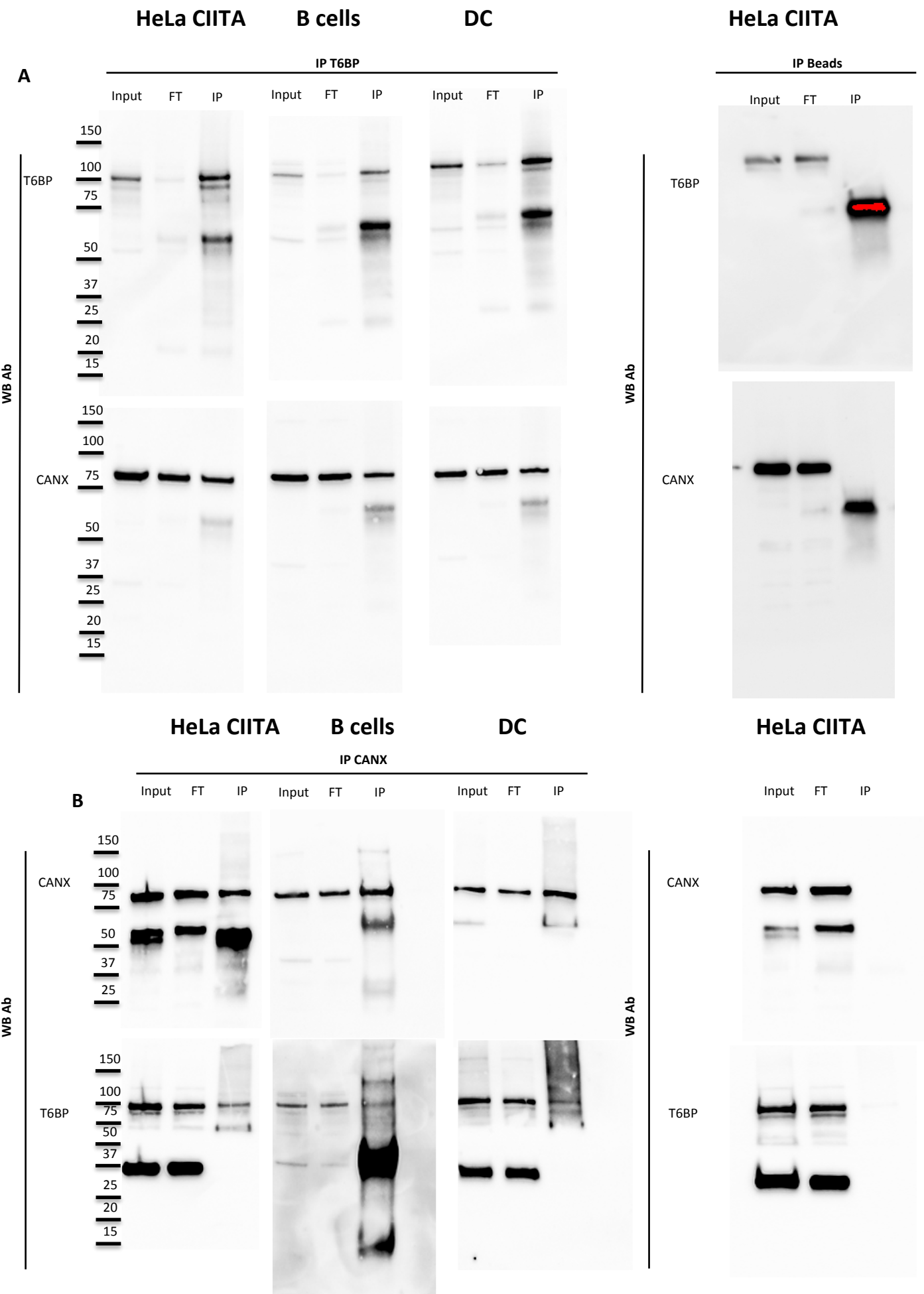

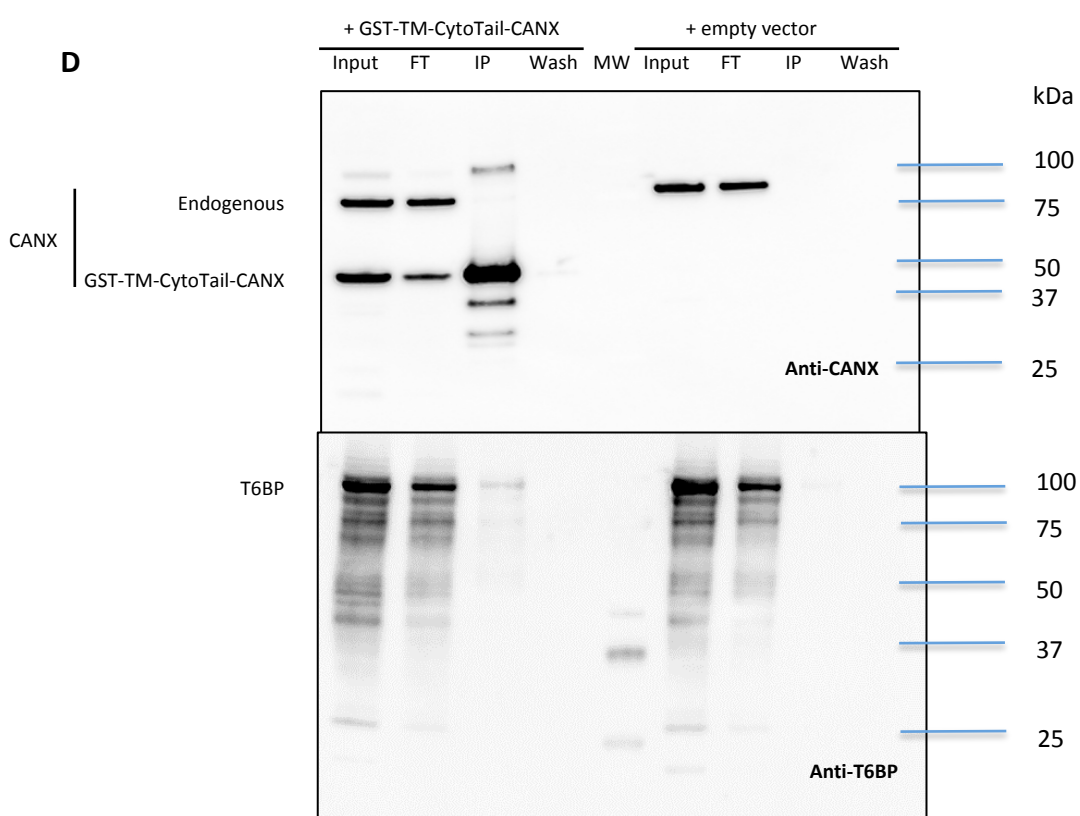

**E**

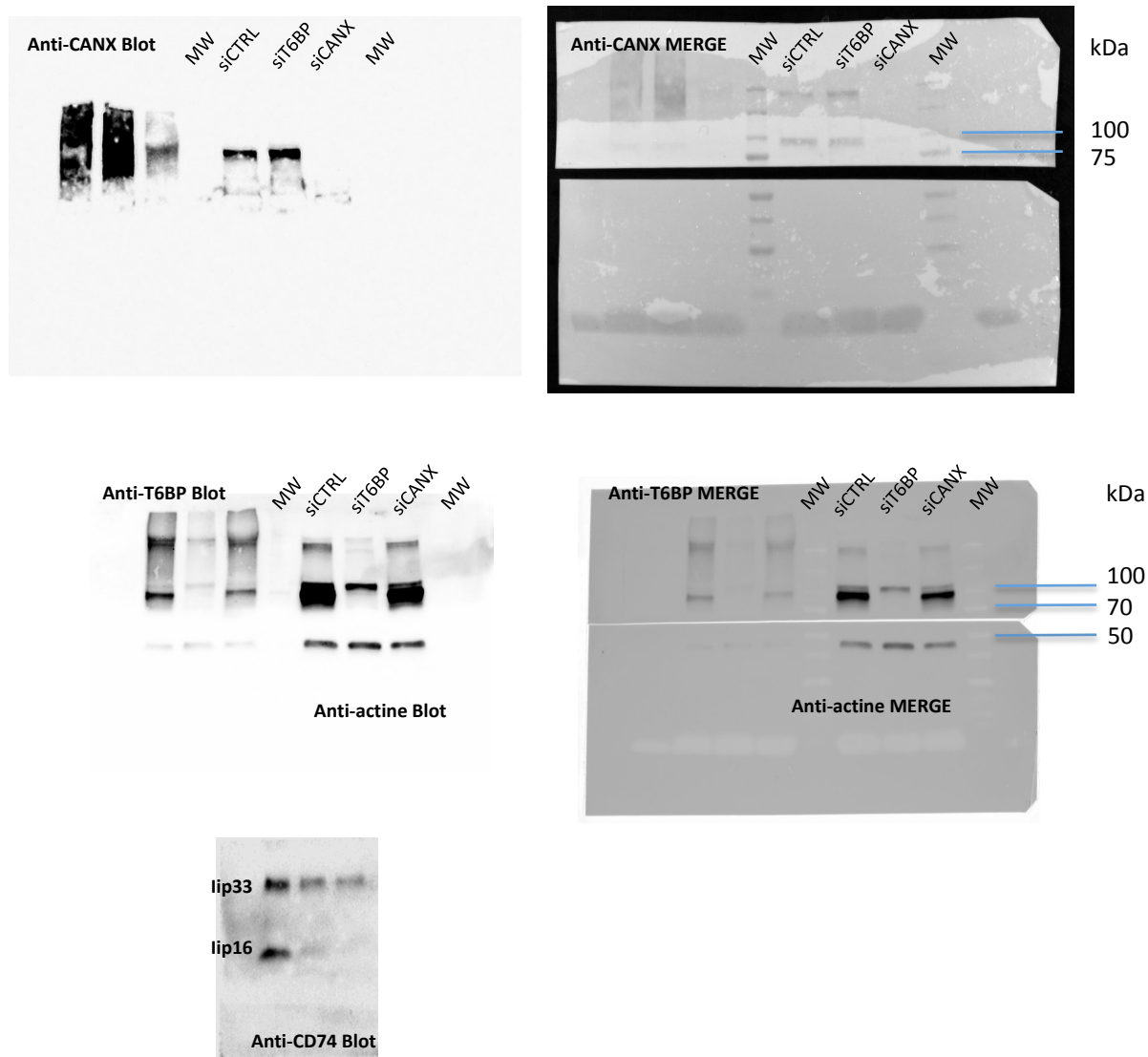

WB data for Figure-7.

Supplement: Supplementary file 7 — Source Data for Figure 7 [file EMBR-23-e55470-s005.pdf]
