# Supplementary material for: The Autophagy Receptor TAX1BP1 (T6BP) improves antigen presentation by MHC‐II molecules
Source: EMBO Rep. 2022 Oct 10;23(12):e55470. doi: 10.15252/embr.202255470 (PMC9724678; doi:10.15252/embr.202255470)
Supplement: Supplementary file 9 [file EMBR-23-e55470-s004.pdf]

EMBO reports

The Autophagy Receptor TAX1BP1 (T6BP) improves antigen presentation by MHC-II molecules.

Gabriela Sarango, Clemence Richetta, Mathias Pereira, Anita Kumari, Michael Ghosh, Lisa Bertrand, Cedric Pionneau, Morgane Le Gall, Sylvie Gregoire, Raphael Jeger-Madiot, Elina Rosoy, Frédéric Subra, Olivier Delelis, Mathias Faure, Audrey Esclatine, Stephanie Graff-Dubois, Stefan Stevanovic, Benedicte Manoury, Bertha Ramirez, and Arnaud Moris
DOI: 10.15252/embr.202255470

Corresponding author(s): Arnaud Moris (arnaud.moris@i2bc.paris-saclay.fr) , Benedicte Manoury (benedicte.manoury@inserm.fr)

Review Timeline:

Transfer from Review Commons:	23rd May 22
Editorial Decision:	21st Jun 22
Revision Received:	30th Aug 22
Editorial Decision:	8th Sep 22
Revision Received:	16th Sep 22
Accepted:	23rd Sep 22

Editor: Achim Breiling

Transaction Report: This manuscript was transferred to EMBO Reports following peer review at Review Commons.

(Note: With the exception of the correction of typographical or spelling errors that could be a source of ambiguity, letters and reports are not edited. Depending on transfer agreements, referee reports obtained elsewhere may or may not be included in this compilation. Referee reports are anonymous unless the Referee chooses to sign their reports.)

Review

1. Evidence, reproducibility and clarity:

Evidence, reproducibility and clarity (Required)

Synopsis

In their manuscript, Pereira et al study the role of TAX1BP1 on the MHCII-dependent antigen presentation. The authors found TAX1BP1 to be necessary for antigen presentation and to affect both quality and quantity of antigens presented on MHCII molecules. The authors examined the effect of the lack of TAX1BP1 (siRNA) on the localization of the MHCII-loading compartments. The authors further showed that silencing TAX1BP1 causes a rapid degradation of the invariant chain (CD74) without altering the expression and internalization kinetics of MHCII molecules. Using an IP-MS method, they also defined Calnexin as an interaction partner of TAX1BP1. Although some of these results are potentially interesting and of interest, in particular for researchers studying the role of immuno-autophagy, the study falls short of revealing any detailed molecular mechanism or showing any physiological significance.

Specific Comments

1.In general, the mechanism of how TAX1BP1 may be involved in antigen presentation is still unclear.

2.The results have been shown only in one cell line, and the observed effects of TAX1BP1 silencing may be cell-specific. Also, the antigens to be presented on the MHCII molecules are already attached to LC3-G120 proteins which does not represent a physiological setting.

3.Instead of using siRNA, CRISPR silencing should have been chosen to silence TAX1BP1. This has become a gold standard and tends to have fewer non-specific effects especially with regard to studying the effect of gene silencing on immunity-related processes.

4.Rescue experiments are missing in general. For example, the reduction of T-cell activation derived from siTAX1BP1 could be reverted by re-expressing a siRNA-resistant version of TAX1BP1.

5.Autophagy-independent role of TAX1BP1 has been explained using a G120A form of LC3 protein. However, it is known that LC3-G120A protein still can accumulate in inclusion bodies or aggresomes and deliver their conjugated viral peptides to autophagosomes. Please consider that repeating these experiments with an ATG7 KO cell line could reveal a true autophagy-independent role. Chemical compounds such as Bafilomycin A1 or Chloroquine could also be used to test overall autophagy effect on the observed phenotypes of TAX1BP1 silencing.

6.The link between TAX1BP1 and Calnexin has been covered only superficially. There needs to be more to tell about them. Additionally, since silencing calnexin will interfere with membrane trafficking, vesicular transport and secretory pathways in the cell, it is not surprising that it is reducing activation of T cells.

7.There are some reports explaining how TAX1BP1 might be involved in endosomal cargo transport (Jongsma et.al., 2016). As the authors observed that TAX1BP1 silencing could lead to mislocalization of MHCII molecules, this effect might be related to its role in vesicular trafficking. This point needs to be clarified and explored further.

8.For each marker protein used for confocal experiments, there is a need to show how their expression is affected by siRNA treatments. For example, in fig 4, how are LAMP-1 and CD63 level affected by siRNA treatments?

9.It is asserted in the text that TAX1BP1 silencing causes a rapid degradation of CD74. However, the pulse-chase experiment is not enough to clarify this point. Changes in mRNA level of CD74 also need to be checked as a consequence of siRNA treatment. Also, the degradation mechanism should be better elucidated. Proteasomal inhibitors (e.g. Velcade) or lysosomal inhibitors (e.g. Bafilomycin A1) should reverse the observed effects.

10.Input controls for each IP experiment need to be provided. For example, we need to know the amount of proteins loaded onto beads in Fig 5, and how silencing TAX1BP1 or protein overexpression changes Calnexin levels in the cytosol or at the cell membrane.

2. Significance:

Significance (Required)

The study is of potential interest but requires substantially more work for impact (see comments above).

3. How much time do you estimate the authors will need to complete the suggested revisions:

Estimated time to Complete Revisions (Required)

(Decision Recommendation)

Between 3 and 6 months

Review #2

1. Evidence, reproducibility and clarity:

Evidence, reproducibility and clarity (Required)

The authors demonstrate that among several autophagy receptors (p62, NDP52, optineurin and TAX1BP1)

only TAX1BP1 silencing decreases intracellular antigen presentation by MHC class II molecules of HeLa-CIITA cells. This affects both antigens that are targeted to autophagosomes via fusion with LC3 and untargeted antigens, but not extracellular peptide presentation by MHC class II molecules. Accordingly, the MHC class II, but not the MHC class I ligandome is changed upon TAX1BP1 silencing. While the location of the MHC class II containing compartment (MIIC) is altered in the absence of TAX1BP1 MHC class II internalization is not affected. Instead, invariant chain (Ii)/CD74 degradation is increased upon TAX1BP1 loss. The authors identify calnexin as an interaction partner of TAX1BP1 and calnexin silencing phenocopies TAX1BP1 loss. From these findings the authors conclude that TAX1BP1 stabilizes CD74 for efficient complex formation with MHC class II molecules, and increased CD74 degradation in the absence of TAX1BP1 compromises MHC class II presentation of intracellular antigens.

The manuscript reports interesting findings but some additional information on the TAX1BP1 interaction with calnexin, especially in professional antigen presenting cells, should be provided.

Major comments:

1.The authors demonstrate that TAX1BP1 silencing alters the peptide ligandome of MHC class II, but not MHC class I molecules. Are there any changes with respect to source of peptides (longevity of source proteins)? Is there any overlap with the source of peptides that were previously described to be influenced by autophagy (Dengjel et al., PNAS 2005)?

2.Interaction of calnexin with TAX1BP1 via its cytosolic portion? Although most domains of calnexin are luminal in the endoplasmic reticulum (ER), around 90 amino acids are located in the cytosol, where they might co-localize with TAX1BP1. Is this domain required for TAX1BP1 interaction?

3.Although the HeLa-CIITA cells are a good model system, it would be important to confirm the role of TAX1BP1 for endogenous antigen presentation by MHC class II molecules in a professional antigen presenting cell. Since the authors already tried to assess monocyte-derived dendritic cells, but elicited their maturation during the investigated manipulations, B cell lines could be tested for dependence on TAX1BP1 for intracellular antigen presentation on MHC class II molecules.

Minor comments:

1.The text should refer to CD4+ T cells always in the same way. Currently, CD4 T cells, CD4superscript+ T cells and CD4+ T cells are in use.

2.The authors describe the GFP moiety of their GFP-TAX1BP1 protein that they use for their immunoprecipitations as bait, but it would probably be more correct to describe the GFP as tag and TAX1BP1 as bait.

2. Significance:

Significance (Required)

The authors unveil an interesting function of TAX1BP1 in intracellular antigen presentation on MHC class II molecules, which provides valuable insights into this important antigen presentation pathway for helper T cell stimulation.

Interesting molecular interactions that influence antigen presentation to helper T cells, comparable to Fletcher

et al., EMBO J 2018.

Interesting for researchers in immunology and infectious diseases.

I have expertise in viral antigen presentation on MHC molecules and autophagy.

3. How much time do you estimate the authors will need to complete the suggested revisions:

Estimated time to Complete Revisions (Required)

(Decision Recommendation)

Between 1 and 3 months

Review #3

1. Evidence, reproducibility and clarity:

Evidence, reproducibility and clarity (Required)

In this manuscript, Moris and colleagues describe a study of TAX1BP1/T6BP, an autophagy receptor, in the context of MHC class II presentation. The authors use HeLa cells modified to express the CIITA for induction of MHC class II expression in all of their studies. The initial rationale for the research is the hypothesis that one or more autophagy receptors (ARs) contribute to the processing and presentation of MHC class II epitope, owing to a previously reported role for autophagy in this process. The authors start by silencing the expression of multiple ARs to determine which, if any, is required for MHC II presentation. Antigen presentation is evaluated using IFNg ELISpot measurements with a T cell line responsive to gag peptide. Using this approach, the authors determine that T6BP1 is the only AR that appears to be required for antigen presentation. The remainder of the study then focuses T6BP1 and its interaction (or lack thereof) with the MHC II compartment and a search for proteins that potentially interact with T6BP1. The overall conclusion put forward by the authors is that T6BP1 modulates the stability of the invariant chain CD74 possibly via direct interaction with calnexin, and that this role impacts the repertoire of peptides loaded onto MHC II complexes and ultimately affects T cell stimulation. This conclusion is supported by measurements of MHC II surface levels, localization of MHC II containing vesicles, a survey of the immunopeptidome, T cell activation assays and MS data on putative T6BP1 interacting proteins.
Overall, the study is of interest and reports on the novel discovery that T6BP1 plays a role in the selection of the peptide repertoire presented by MHC II. The experiments presented are mostly well conducted and adequately controlled. The key conclusion that T6BP1 is required for adequate peptide presentation of an endogenously expressed protein (as opposed to soluble peptide added to the system) are well supported, though the presentation of the data can be improved in this regard (see below for specifics). However, the manuscript falls well short of providing a clear mechanism by which T6BP1 impacts MHC II presentation. First, in well conducted immunofluorescence studies, the authors show that T6BP1 does not colocalize with

the MHCII compartment. A direct role in CD74 degradation is therefore quite doubtful. Intriguingly, the authors did not find T6BP1 to colocalize with LC3, even though the proteins are known to interact in autophagy. Collectively, immunofluorescence data are of interest for showing a change, albeit moderate, in the spatial distribution of MHC II, LAMP-1, lysotracker and CD63 containing vesicles and of the colocalization of MHC II with lysotracker and CD63, suggesting a difference in trafficking/maturation of the MHCII compartment.

How T6BP1 would impact the MHCII compartment is unclear. The presentation of antigen is impaired in T6BP1 silenced cells regardless of whether the antigen is directed to the autophagy pathway or not. This suggest that T6BP1 may not directly contribute to antigen processing/digestion, rather that it could more generally impact MHCII loading and/or trafficking (data to support this show that MHCII levels are slightly higher in T6BP1 silenced cells, in slight contradiction with the proposed immaturity of MHCII vesicles that are repositioned closer to the nucleus).

In search for a mechanistic explanation, the authors resorted to immune precipitation of GFP-fused T6BP1 to identify potential interacting proteins. Among the many proteins pulled down in this approach (more than 100), the authors focused on calnexin because of a previously reported action of calnexin in degrading CD74. This is a justifiable choice, even if calnexin was among many candidates, the majority of which may not be reliable T6BP1 interacting partners. In this regard, it would have been very helpful to co-stain cells for calnexin and T6BP1, or calnexin and CD74, to increase confidence in direct interactions. This could be particularly pertinent to demonstrate that the absence of T6BP1 could decrease calnexin colocalization with CD74, which would explain increased degradation of the invariant chain when T6BP1 is silenced.

Generally, the evidence that T6BP1 and calnexin interact is scant and this conclusion is solely derived from the IP experiment, with no confirmatory assays of direct protein-protein interaction. Of note, the IP for GFP alone (Fig7A) shows substantial calnexin pull-down, suggesting that the presence of calnexin in the GFP-T6BP1 IP is not specific. This would not be surprising, given calnexin's role as an ER chaperone that interacts with many proteins. The authors would do well to strengthen the proposed link between T6BP1/Calnexin/CD74 by performing additional experiments to show a specific and meaningful interaction between T6BP1 and calnexin.
Overall, the study remains primarily descriptive, with no insight into how T6BP1 affects the selection of the immunopeptidome (is this an indirect effect, given that T6BP1 does not seem to localize with the MIIC?). A direct link between T6BP1 and calnexin is tenuous, and the effect of calnexin on CD74 degradation had previously been described, leaving the proposed link from T6BP1 to CD74 and peptide loading neither certain nor entirely novel. The key would be to strengthen data on the interaction between T6BP1 and CD74, since this is the authors decided to focus on.

With regards to data presentation and analysis, I would have the following comments and suggestions for improvements:

1)In Fig1B, T cell activation seems quite modest in response to gag transfection, a positive control for maximal stimulation would have been helpful. The normalization of the data in the right panel should be described - were the absolute counts between experiments very divergent or could they be combined without normalizing? The symbols for statistics should be better described - what comparisons to these refer to (vs. CTRL?)

2)In Fig1D, the authors should show absolute counts to show how the different Gag versions transfected compared with each other in mediating activation of T cells. The way data are normalized in the current figure do not allow the reader to determine if targeting Gag to the autophagy pathway vs. preventing this has a significant effect on its presentation and on T cell activation.

3)In Fig2, the mock transfections serve very little purpose as currently analyzed. It would have been much more informative to have two separate siCTRL and two siT6BP1 measurements to compare within and across conditions (is variability between siCTRL and siT6BP1 larger than within either condition?). At a minimum, the authors should show how the siCTRL and siT6BP1 each compare to MOCK1 and MOCK2.

4)Fig7A, the authors extrapolate from the strength of GFP vs. GFP-T6BP1 blotting that there was more GFP than fusion protein in the pull-downs, and then extrapolate that calnexin was 100-fold increased in the fusion IP. This is making the assumption that the GFP antibody binds equally well to GFP as to the fusion protein, which is not necessarily true. The calnexin blot shows very clear presence of the protein in the GFP-only IP. These data should be interpreted with more caution.

Finally, the authors provide a comprehensive and interesting discussion of their data. While the strength of their conclusions may be somewhat overstated, the data are of interest, showing a novel aspect of T6BP1's function, albeit with no precise explanation for the observed effects.

2. Significance:

Significance (Required)

This study reports on a novel observation that T6BP1 has an impact on MHC II antigen presentation, suggesting an effect on peptide loading/selection, with a speculative explanation that T6BP1 may interact with calnexin, known to affect CD74 stability and degradation.
In the context of better understanding how MHC II presentation is regulated, this study provides a new avenue for further exploration. The data are not entirely conclusive as to the mechanism by which T6BP1 impacts MHCII presentation, and the significance of the manuscript therefore is limited. The data could be strengthene with additional experiments to support a direct interaction between T6BP1 and calnexin.

Referees cross-commenting

I largely concur with comments made by reviewer's 1 and 2.
I do think that the use of siRNA is adequate - if sufficiently controlled, and that the authors would not need to repeat their studies with CRISPR KO cells at this point. In this respect, reviewer 1 makes a useful suggestion to use an siRNA insensitive transgene to show that the knockdown phenotype can be reversed by 'rescue experiments'. That said, I do not see this as a critical weakness. Rather, the lack of mechanistic insight, and - as pointed out by the two other reviewers - the fact that the authors used only a single model cell line (HeLa CIITA) in their study are the key points that should be addressed to increase significance.

3. How much time do you estimate the authors will need to complete the suggested revisions:

Estimated time to Complete Revisions (Required)

(Decision Recommendation)

Between 3 and 6 months

We thank all reviewers for thoughtfully comments and suggestions. We appreciate that the manuscript is now greatly improved. **The original comments from the reviewers are in bold** and our answers below.

Reviewer #1:

We thank reviewer-1 for fruitful comments and highlighting that our results are "interesting and of interest, in particular for researchers studying the role of immuno-autophagy". Our work reveals a novel function of autophagy receptors in particular TAX1BP1 in the presentation of MHC-II-restricted viral antigens to CD4+ T cells.
To decipher the underlying molecular mechanisms, in T6BP-silenced cells, we analysed various steps of MHC-II molecule trafficking, loading and functions. Using an MS-based immunopeptidomic approach, we demonstrate that T6BP expression has a major influence on the diversity and quality of the MHC-II ligands, which is probably a direct consequence of the exacerbated degradation of the invariant chain (Ii, CD74) that we observe in T6BP-silenced cells. CD74 is indeed a major actor in the biology of MHC-II molecules dictating the folding of the αβHLA-heterodimer, their trafficking in late endosomes and the affinity of peptides loaded onto MHC-II molecules. Importantly, the stability of CD74 is itself regulated by the ER chaperon calnexin (CANX). Consequently, we performed imaging of intracellular vesicles (in particular MHC-II+ compartments) and we studied the fate of molecules (HLA-DM, Ii/CD74) involved in MHC-II molecules loading and trafficking. We observe that TAX1BP1 silencing influence the stability of αβHLA heterodimers, (in the revised ms), the trafficking of late endosome (including MHC+ HLA-DM+ vesicles), lysosome and autophagosomes. Importantly, in an MS-based interactome, we identified CANX as a partner of T6BP. Thereafter, since CANX is also known to bind CD74, for this first description of the role of TAX1BP1 in antigen presentation, we decided to focus on the role of CANX, to provide insights on one of the mechanisms of action of T6BP. We readily demonstrate, in this revised version of the ms, that T6BP interacts with CANX in various professional antigen-presenting cells. Importantly, we show that T6BP/CANX interaction requires the cytosolic tail of CANX. Finally, we provide the first direct demonstration that in human cells, CANX-silencing induces CD74 degradation and inhibits MHC-II-restricted antigen presentation, thus replicating the functional consequences of T6BP silencing.

1. In general, the mechanism of how TAX1BP1 may be involved in antigen presentation is still unclear. We propose that TAX1BP1 acts at least at two distinct levels on MHC-II loading: 1) the positioning of late endosomes including the MIIC, thus influencing MHC-II molecule maturation and antigen degradation and 2) the function of CANX as a key component of the MHC-II loading complex in the ER.

1) In this revised version, we show that the repositioning of the MIIC leads to the generation of unstable MHC αβ heterodimers which is most likely a direct consequence of CD74 exacerbated degradation. Importantly, we provide evidences that CD74 expression can be recovered when treating cells with chloroquine (Fig5D) and other cathepsin inhibitors (not shown). How T6BP dictates the traffic of the MIIC remains to be determined but might be reminiscent of its action on endosomal trafficking (Jongsma et al., 2016).
2) In the present manuscript, we decide to focus on CANX that we identified as a protein partner of T6BP in our interactome because 1) it has been shown that CANX stabilizes CD74, in the ER, and thus favours the formation of CD74-MHC complexes. 2) we show that silencing of CANX recapitulates the observations made with T6BP-silenced cells. As suggested by the reviewers, to strengthen this point, we now provide insights in the molecular interactions between TAX1BP1 and CANX and new results supporting the physiological relevance of our findings. Using biochemical (co-IP) and proximity ligation assays, we now provide strong evidence demonstrating that TAX1BP1 interacts with Calnexin. We show that TAXBP1 interacts with CANX in non-professional and in professional APC such as B cells and dendritic cells. Finally, using IP, we show that TAXBP1 binds the cytosolic tail of CANX, which is known to regulate CANX functions.

2. The results have been shown only in one cell line, and the observed effects of TAX1BP1 silencing may be cell-specific. As suggested by the reviewer, in order to strengthen our observations, focusing on TAX1BP1 and CANX interplay, using IP, we now show in two different types of immune cells, B and dendritic cells (DC) that TAX1BP1 interacts specifically with CANX. This is now highlighted in the text page 14 and in (Fig 7A and B): "*To demonstrate that T6BP/CANX interaction is not restricted to Hela-CIITA cells, we then immunoprecipitated endogenous T6PB and CANX from two professional APCs, namely B cells and DC. As in Hela-CIITA, we revealed in the anti-T6BP and anti-CANX IP fractions, of both B cells and DC, the presence of CANX and T6BP, respectively (Fig 7A-B).*"

Also, the antigens to be presented on the MHCII molecules are already attached to LC3-G120 proteins which does not represent a physiological setting. As mentioned by the reviewer, in our work we used various model antigens such as Gag-LC3 (Fig 1, to target Gag into autophagosome) side by side with Gag-LC3-G120A (Fig 1, as negative control for autophagosome targeting) but we also used native Gag antigen from HIV-1 (Fig 1) and native pp65 antigen from HCMV (Fig 1E and 7F). In all cases we observed a strong influence of TAX1BP1 silencing on antigen-specific CD4+ T cell activation. In addition using immunopeptidomic experiments, we analysed the influence of T6BP silencing on the global repertoire of self naturally presented peptides by HLA-II molecules. We observed that "*1349 peptides (representing 25% of the peptides) were presented by MHC-II molecules exclusively in HeLa-CIITA cells expressing T6BP (Fig 3A, right panel, siCTRL). Remarkably, in the absence of T6BP expression, 2198 new MHC-II ligands (40% of the peptides) were identified (Fig 3A, right panel, siT6BP). Overall, only 35% of the peptides (1914 peptides) were shared between control and the T6BP-silenced conditions (Fig 3A, right panel).*" We concluded that "*the absence of T6BP has a pronounced and dramatic influence on the repertoire of peptides presented by MHC-II molecules.*" We believe that these results demonstrate that the action of T6BP on antigen presentation is not limited to a single model antigen.
Nonetheless for clarity, the text page 6 has been modified: "*Importantly, in our previous work we demonstrated that newly synthetized Gag is processed in an autophagy independent manner both in monocyte-derived DCs (MDDCs) and in HeLa-CIITA cells (Coulon et al., 2016). This was shown by using productively infected MDDCs and Gag-cDNA-transfected HeLa-CIITA cells treated with various drugs influencing autophagy (e.g. 3-MA, Spautin-1 and Torin-1), shRNA targeting LC3 or overexpressing a trans-dominant mutant of Atg4B (Atg4BC74A), which blocks the formation of autophagosomes. Using the same tools, we also showed that the fusion with LC3 (Gag-LC3) targets Gag to autophagosome-mediated degradation leading to an enhancement of CD4^{+} T cell activation. Gag-LC3$_{G120A}$ was also used as a negative control for autophagy-dependent degradation, as the G120A mutation in the C-terminus of LC3 abolishes the lipidation and incorporation of LC3 in the nascent membranes of autophagosomes, thus preventing Gag targeting into autophagosomes. Finally, we showed that upon Gag-LC3$_{G120A}$-transfection, Gag antigens are processed in an autophagy independent manner (Coulon et al., 2016).*"

3. Instead of using siRNA, CRISPR silencing should have been chosen to silence TAX1BP1. This has become a gold standard and tends to have fewer non-specific effects especially with regard to studying the effect of gene silencing on immunity-related processes.
We fully agree that the CRISPR-Cas9 technology provides powerful tools for gene therapies and to generate KO cell lines for the study of molecular mechanism. However, it also has some limitations such as off-target effects and more importantly the development/existence of compensatory/redundant mechanisms when knocking-down single genes. These limitations have been particularly highlighted in the study of selective autophagy receptors and their involvement in mitophagy. Using siRNA and HeLa cells, Wong *et al.* demonstrated that Optineurin (OPT) plays a major role in mitophagy (Wong *et al.,* 2014). In contrast using OPT KO HeLa cells, Lazarou *et al.* failed to recapitulate theses finding (Lazarou *et al.,* 2015). Only when using triple OPT/NDP52/TAX1BP1 KO HeLa cells could they indeed confirm that OPT is involved in mitophagy (Lazarou *et al.,* 2015). The authors concluded that OPT and NDP52 are redundant. Stable invalidation of OPT forces the cells to adapt and use redundant mechanisms to survive. Short-term invalidation of protein expression, using siRNA, in contrast allowed, Wong *et al.* to pinpoint the function of OPT.
Nonetheless using in-house TAX1BP1 KO HeLa-CIITA cells and TAX1BP1 KO HeLa, from Lazarou *et al.*, transfected with CIITA (to induce the expression of the MHC-II molecules), we intended to further study the role of TAX1BP1 in MHC-II-restricted antigen presentation. However, as in Lazarou *et al*, we failed in showing an influence of TAX1BP1 on antigen presentation using these single TAX1BP1 KO HeLa-CIITA cell lines (not shown). We extended this work to the triple OPT/NDP52/TAX1BP1 KO HeLa (also from Lazarou *et al*) in which we transfected CIITA. This was an obvious choice since NDP52 is a close homolog of TAX1BP1. Again the triple OPT/NDP52/TAX1BP1 KO HeLa-CIITA did not exhibit significant impairments in antigen presentation (not shown). This might be explained by redundant functions potentially exerted by p62, NBR1 and/or Tollip. Thinking that it might be a never-ending search for redundant autophagy receptor KO, as Wong *et al.*, we decided to focus on the siRNA approach and screen additional siRNA. Using 6 siRNA in total from two different providers and targeting different sequences (in exons and introns), we demonstrate that TAX1BP1 influence antigen presentation.

4. Rescue experiments are missing in general. For example, the reduction of T-cell activation derived from siTAX1BP1 could be reverted by re-expressing a siRNA-resistant version of TAX1BP1.
We agree with reviewer-1 that this might have been an interesting result. However, these rescue experiments require multiple parameters to align in order to draw conclusions, in particular a triple transfection. First, the HeLa-CIITA cells should be transfected with the scramble or anti-TAX1BP1 siRNAs and then with a plasmid encoding an siRNA-insensitive TAX1PB1. Finally, these cells must be transfected with the plasmid encoding the

antigen. A critical point is to reach in CTRL and anti-TAX1BP1 siRNA treated cells similar levels of: 1) siRNA insensitive TAX1BP1 expression and 2) antigen-positive cells. Which is not that trivial following a triple transfection that also impacts cell viability. We intended to perform these experiments and indeed observed great variability of transfection rates, cell death, even when playing with the kinetics of the triple transfection prior co-culture with the T cells. Finally, these preliminary experiments were inconclusive. We decided not to pursue in this direction in particular since reviewer-3, did not see the absence of rescue experiments has a "critical weakness".

5. Autophagy-independent role of TAX1BP1 has been explained using a G120A form of LC3 protein. However, it is known that LC3-G120A protein still can accumulate in inclusion bodies or aggresomes and deliver their conjugated viral peptides to autophagosomes. Please consider that repeating these experiments with an ATG7 KO cell line could reveal a true autophagy-independent role. Chemical compounds such as Bafilomycin A1 or Chloroquine could also be used to test overall autophagy effect on the observed phenotypes of TAX1BP1 silencing.

The autophagy-independent effect of TAX1BP1 was explored using Gag-LC3-G120A, as mentioned by rewiewer-1, but also using the native Gag protein (Fig.1D). Importantly, in our previous work we demonstrated that newly synthetized Gag is processed in an autophagy independent manner both in MDDC and HeLA-CIITA cells (Coulon et al, 20016). These was highlighted using productively HIV-infected MDDC and Gag-cDNA-transfected HeLA-CIITA cells treated with various drugs influencing autophagy (e.g. 3-MA, Spautin-1 and Torin-1), shRNA targeting LC3 or overexpressing a trans-dominant mutant of Atg4B (Atg4BC74A), which blocks the formation of autophagosomes (Coulon et al, 20016). As for Gag, we also showed that the overexpression of Atg4BC74A by Gag-LC3-G120A-transfected Hela-CIITA or -transduced DC did not affect their capacity to activated Gag-specific CD4+ T cells (Coulon et al, 20016). Overall, our published work shows that upon cDNA expression of Gag and Gag-LC3-G120A, Gag antigen processing and presentation is independent of autophagy. Thereafter, the fact that TAXBP1 silencing also inhibits the capacity of HeLA-CIITA cells transfected with Gag cDNA to activate CD4+ T cells strongly suggest that TAXBP1 also influences autophagy-independent MHC-II-restricted antigen presentation. In order to respond to the reviewer's comment, we further highlighted in the text page 6 the fact that Gag processing is indeed independent on autophagy: "*Importantly, in our previous work we demonstrated that newly synthetized Gag is processed in an autophagy independent manner both in monocyte-derived DCs (MDDCs) and in HeLa-CIITA cells (Coulon et al., 2016). This was shown by using productively infected MDDCs and Gag-cDNA-transfected HeLa-CIITA cells treated with various drugs influencing autophagy (e.g. 3-MA, Spautin-1 and Torin-1), shRNA targeting LC3 or overexpressing a trans-dominant mutant of Atg4B (Atg4BC74A), which blocks the formation of autophagosomes. Using the same tools, we also showed that the fusion with LC3 (Gag-LC3) targets Gag to autophagosome-mediated degradation leading to an enhancement of* $CD4^+$ *T cell activation."* as well as Gag-LC3-G120A: " *Finally, we showed that upon Gag-LC3G120A-transfection, Gag antigens are processed in an autophagy independent manner (Coulon et al., 2016).*"

6. **The link between TAX1BP1 and Calnexin has been covered only superficially. There needs to be more to tell about them.**

As suggested by Reviwer-1, we investigated further the link between TAX1BP1 and CANX. This is now highlighted page 14 and (Fig 7A-C): "*In HeLa-CIITA cells, we further confirmed the specific interaction between T6BP and CANX using two different techniques: 1) by immunoprecipitation of endogenous T6BP and endogenous CANX, we revealed that the IP fractions of anti-T6BP and anti-CANX IPs contained CANX and T6BP, respectively (Fig 7A-B); 2) by proximity ligation assay (PLA) on control cells and siT6BP-transfected cells a specific T6BP/CANX interaction was detected only in cells expressing T6BP (Fig 7C). To demonstrate that T6BP/CANX interaction is not restricted to Hela-CIITA cells, we then immunoprecipitated endogenous T6PB and CANX from two professional APCs, namely B cells and DC. As in Hela-CIITA, we revealed in the anti-T6BP and anti-CANX IP fractions, of both B cells and DC, the presence of CANX and T6BP, respectively (Fig 7A-B)."* Taken together, these results strongly support that CANX interacts with T6BP directly or as part of a larger protein complex.

In addition, we characterize on CANX the domain of interaction with TAX1BP1, these results are described page 15 and in (Fig 7F): "*In order to characterize the molecular interactions between CANX and T6BP, we then engineered a construct containing solely the transmembrane domain and the cytosolic tail of CANX fused to a GST tag. Since T6BP expression is mainly cytosolic, we reasoned that T6BP and CANX might interact through the cytosolic tail of CANX. The GST-TM-cytosolic Tail construct was transfected in Hela-CIITA cells and immunoprecipitated using anti-GST antibodies. Western blotting revealed that T6BP is co-immunoprecipitated with anti-GST in cells transfected with GST-TM-cytoTail and not in mock-transfected cells (Fig 7D). Taken together, we demonstrate here that T6BP interacts with CANX through its cytosolic tail.*" We propose that

through binding to the cytosolic tail of CANX, T6BP promotes the interaction of CANX with CD74 leading to CD74 stabilization.

Additionally, since silencing calnexin will interfere with membrane trafficking, vesicular transport and secretory pathways in the cell, it is not surprising that it is reducing activation of T cells.
As mentioned by Reviewer-1, CANX has been shown to participate in multiple cellular pathways from protein quality control to the regulation of Ca2+ flux. Thereafter, we performed a variety of controls on siCANX treated cells:
- Using WB and FACS, we monitored the expression levels of anti-HLA- DR and -DM molecules. It did not reveal any significant differences between siCTRL- and siCANX-treated cells (not shown).
- In our IFNγ-ELispot assay, siCTRL- and siCANX-treated cells were loaded exogenously with the cognate peptide to compare their capacity to activate antigen-specific CD4+ T cells (Fig 7F). Again here, we did not notice significant differences.
The absence of significant differences between control and siCANX treated cells might be explained by the redundant functions of CANX with calreticulin or other chaperones. Indeed, Molinari M *et al.* for instance showed that depending on the glycoproteins investigated, individual loss of CANX or calreticulin did not affect tightness of ER quality control (Molinari et al, 2004). We believe that these controls and published works allow to conclude that the reduction in T cell activation observed upon CANX silencing highlights a specific defect in the antigen processing routes and not just an overall perturbation of the cell. Page 14 a sentence was added to highlight this point: *"To this end, we screened several siRNA targeting Calnexin and identified an siRNA whose transfection, lead to strong decrease of CANX expression (siCANX) (Fig S7E), without affecting neither HLA-DR and -DM expression levels nor the viability of transfected Hela-CIITA cells (not shown)."*

7. There are some reports explaining how TAX1BP1 might be involved in endosomal cargo transport (Jongsma et.al., 2016). As the authors observed that TAX1BP1 silencing could lead to mislocalization of MHCII molecules, this effect might be related to its role in vesicular trafficking. This point needs to be clarified and explored further.
We suggest here that CANX is a key factor involved in T6BP-mediated regulation of MHC-II loading that strongly influences the repertoire and affinity of presented peptides. However, we do not exclude that other cellular factors or pathways also participate in T6BP action on MHC-II molecule trafficking. The text, page 18, has been modified to enriched the discussion on the role of T6BP in vesicle trafficking: "*Jongsma et al showed that the ER ubiquitin ligase RNF26 interacts with p62 (SQSTM1) and that it employs a ubiquitin-based communication with Tollip, T6BP, or EPS15 to cluster recycling, early and late endosomes in so-called perinuclear clouds. In the model, deubiquitination of p62 by the DUB UPS15 leads to the release of the vesicles for maturation* (Jongsma et al., 2016). *The authors showed that the silencing of RNF26 leads to the dispersion of EEA1+ and CD63+ vesicles, in the cytoplasm of the cells. They also show that the silencing of T6BP has a broad influence on the shape of cells and leads to the dispersion of the cloud of CD63+ vesicles. Our observations also highlight that T6BP-silencing influences CD63+ vesicles, however our results indicate that CD63+ vesicles are maintained in the perinuclear region. We confirmed these observations using lysotracker and LAMP-1 markers of acidified and late endosomes respectively. The different behaviour of CD63+ vesicles in siT6BP-treated cells might be due to the expression of the HLA locus driven by CIITA, in our model of APCs. Nonetheless, our work confirms the involvement of T6BP in the traffic of endosomes. In addition, we found, in the interactome study of T6BP, the DUB UPS15. Interestingly, although T6BP affects EEA1+ early endosomes perinuclear localization, we excluded the possibility that the effect of T6BP on MHC-II-restricted antigen presentation relies on early endosomal defect since EEA1+ vesicles do not contain MHC-II molecules in our APCs. Overall, we show that T6BP impacts the trafficking of MHC-II-rich compartments that bear hallmarks of the MIIC, namely HLA-DM, CD63+ and LAMP-1. More globally T6BP-silencing seems to affect the cellular localization of acidified vesicular compartments corresponding to late endosome, lysosomes and as already demonstrated autophagosomes (Petkova et al., 2017), without significantly reducing the expression levels of the various markers. Accumulating evidences suggest that the positioning of intracellular vesicles controls their functions (Neefjes et al, 2017) and intravesicular pH in particular (Johnson et al, 2016)."* For this manuscript, we decided to focus on a second aspect of T6BP functions in antigen presentation that is the degradation of CD74 and the interactions with CANX.

8. For each marker protein used for confocal experiments, there is a need to show how their expression is affected by siRNA treatments. For example, in fig 4, how are LAMP-1 and CD63 level affected by siRNA treatments?
As suggested by reviewer-1 we included in the revised version the quantification of the expression levels of each marker used in the study. To this end for all markers (LAMP-1, Lysotracker, CD63, EEA1, HLA-DR, -DM and CD74) (Fig S5, S6 and Fig 4), we analysed the MFI using confocal imaging that we completed with WB (Fig 5)

and FACS (Fig 2) for the most relevant proteins of the study (HLA-DR, -DM and CD74). A sentence has been in added in the results section, page 11, to highlight this point: *"Also note that overall, T6BP-silencing did not reduce significantly the expression levels of the various markers analysed using flow cytometry analysis (Fig 2), WB (Fig 4 and 5) and IF (Fig S5C-H)."* and in the discussion, page 18: "*More globally T6BP-silencing seems to affect the cellular localization of acidified vesicular compartments corresponding to late endosome, lysosomes and as already demonstrated autophagosomes (Petkova et al., 2017), without reducing significantly the expression levels of the various markers.*"

9. It is asserted in the text that TAX1BP1 silencing causes a rapid degradation of CD74. However, the pulse-chase experiment is not enough to clarify this point. Changes in mRNA level of CD74 also need to be checked as a consequence of siRNA treatment. Also, the degradation mechanism should be better elucidated. Proteasomal inhibitors (e.g. Velcade) or lysosomal inhibitors (e.g. Bafilomycin A1) should reverse the observed effects.

We thank reviewer-1 for suggesting to include these important controls. We now provide evidences that in contrast to the mRNA of TAX1BP1, CD74 mRNA levels are not affected by the transfection of siRNAs targeting TAX1BP1 (Fig S6D and E). In addition, we show that drugs affecting lysosomal acidification (Fig 5D) and protease activities (not shown) allow reversing the effect of TAX1BP1-silencing on CD74 expression levels. In contrast inhibiting proteasomal degradation did not rescue CD74 expression (Fig 5D). The following paragraph (page 12) and new results have been added (Fig 5D and S6D and E): "*We then asked at which level CD74 expression was affected, meaning at the RNA or protein levels. To this end, we first used RT-qPCR to monitor CD74 and T6BP mRNA relative quantities in control and T6BP-silenced HeLa-CIITA cells. As expected T6BP mRNA levels were strongly reduced in siT6BP-treated cells (Fig S6D). In contrast, mRNA levels of CD74 were not influenced by T6BP silencing (Fig S6E). We next analysed whether CD74 expression could be reversed, in siT6BP-silenced cells, upon treatment with drugs inhibiting lysosomal or proteasomal degradation, using Chloroquine or Epoxomicin, respectively. As expected, treatment of control cells with Chloroquine, led to the accumulation of Iip33 and Iip16, and allowed the detection of the intermediate degradation fragment Iip22 (Fig 5D). Remarkably, when compared to control untreated cells, Chloroquine treatment allowed a strong recovery of both Iip33 and Iip16 detection in siT6BP-treated cells (Fig 5D). In contrast, Epoxomicin treatment did not affect the expression levels of CD74 (Fig 5D).*"

10. Input controls for each IP experiment need to be provided. For example, we need to know the amount of proteins loaded onto beads in Fig 5,

We thank the reviewer-1 for these suggestions. Input IP controls have been added to the IP data of the new Fig 7 demonstrating the interaction of endogenous T6BP with endogenous CANX. Concerning the Fig 5, the amount of protein loaded onto the beads as been quantified and is strictly identical for each conditions. This is now stated in the M&M section. However, as it is a metabolic labelling, we do not show the input fraction.

and how silencing TAX1BP1 or protein overexpression changes Calnexin levels in the cytosol or at the cell membrane.

In Fig 7E and S7F, we observed that silencing of TAX1BP1 did not influence CANX expression levels. This is now stated page 14: "*Note that neither siT6BP nor siCANX transfections lead to an increase expression of CANX or T6BP respectively (Fig 7E and S7E).*" Using confocal imaging, we did not notice a substantial modification of CANX cellular localisation in siT6BP-treated cells (not shown).

Following over expression of T6BP-GFP or GFP the expression levels of CANX in HeLa-CIITA cells were analysed in the cellular extract (using WB) and we did not notice any difference (data not shown). More importantly, the interaction of CANX and TAX1BP1 is now confirmed in various APC, studying endogenous proteins.

Significance: We demonstrate here that TAX1BP1 is necessary for antigen presentation and affecting both the quality and the repertoire of MHC-II ligands. We show that T6BP affects both the traffic of the MIIC and the folding and stability of MHC-II molecule. We provide strong evidences that the latter is regulation by TAX1BP1 interaction with CANX that stabilises the invariant chain CD74.

Reviewer #2:

We thank Reviewer-2 for the comments/suggestions and highlighting that our work unveils an interesting function of TAX1BP1 in intracellular antigen presentation on MHC class II molecules, and provides valuable insights into this important antigen presentation pathway for helper T cell stimulation.

The manuscript reports interesting findings but some additional information on the TAX1BP1 interaction with calnexin, especially in professional antigen presenting cells, should be provided.
We now provide additional data on the interaction between TAX1BP1 and Calnexin in particular in antigen presenting cells, please see below.

Major comments:

1. The authors demonstrate that TAX1BP1 silencing alters the peptide ligandome of MHC class II, but not MHC class I molecules. Are there any changes with respect to source of peptides (longevity of source proteins)?
We thank reviewer-2 for this question. We now include in the ms a cell component enrichment analysis using Funrich software comparing siCRTL and siT6BP treated cells. We show the bulk of MHC-II-ligand source proteins is provided by the same cellular fractions mainly membranes, secretory pathways and the cytosol. However, the expression of T6BP might also favour the presentation of peptide derived from the nucleus. The following paragraph has been added page 9 and the results are presented in figure S2B: "*We then asked whether T6BP expression might affect the source of peptides, meaning the set of proteins supplying peptides for MHC-II loading. To this end, we submitted the LC-MS/MS data to cell component enrichment analysis using Funrich software (Pathan et al, 2015). As expected from previous work (Dengjel et al., 2005; Marcu et al, 2021), according to Funrich annotation and as compared with the human proteome, in both siCTRL and siT6BP treated cells, the MHC-II-ligand source proteins were enriched in cellular fractions belonging to membranes, secretory pathways (e.g. exosomes and vesicles) but also to the cytosolic compartment of the cell (Fig S2B). In siCTRL treated cells MHC-II source proteins were also augmented in nuclear fractions, which was not the case for siT6BP-transfected cells. In contrast, MHC-II-ligand source proteins belonging to the cytoplasm were enriched in siT6BP-treated cells (Fig S2B). Overall, this analysis shows that the bulk of MHC-II-ligand source proteins is provided by the same cellular fractions mainly membranes, secretory pathways and the cytosol. However, the presence of T6BP might also favour the presentation of peptides derived from the nucleus.*"
However, we believe that these differences in the source proteins cannot by themselves explain the influence of T6BP on MHC-II-restricted antigen presentation. A sentence has been added page 17 to highlight our point of view: "Although we observed, using functional enrichment analysis (Funrich, Fig S2), a slight influence of T6BP silencing on the cellular distribution of the source protein of MHC-II ligands, this cannot by itself explain the effects of T6BP on the peptide repertoire. In particular since, T6BP expression also dictates the relative affinity of pepides presented by MHC-II molecules."

Is there any overlap with the source of peptides that were previously described to be influenced by autophagy (Dengjel et al., PNAS 2005)? Dengjel et al. used a different experimental system to study the influence of autophagy on MHC-II peptide presentation, meaning an Epstein-Barr Virus (EBV) transformed human B-lymphoblastoid cell lines in which autophagy was induced by starvation. Owing to the intrinsic variability of the MHC-II ligandome when analysing simultaneously the ligandome of two samples derived from the same cell line (please see the work of Alvaro-Benito et al, 2018 and ours Fig 3), we believe that comparing the two data sets derived using two model systems will not bring much information except if we repeat the experiment with the same cells. Still this is interesting point that could be addressed in the future.

2. Interaction of calnexin with TAX1BP1 via its cytosolic portion? Although most domains of calnexin are luminal in the endoplasmic reticulum (ER), around 90 amino acids are located in the cytosol, where they might co-localize with TAX1BP1. Is this domain required for TAX1BP1 interaction?
We thank reviewer-2 for this important suggestion. In the revised ms, we now characterize on CANX the domain of interaction with TAX1BP1, these results are described page 14 and in (Fig 7F): "*In order to characterize the molecular interactions between CANX and T6BP, we then engineered a construct containing solely the transmembrane domain and the cytosolic tail of CANX fused to a GST tag. Since T6BP expression is mainly cytosolic, we reasoned that T6BP and CANX might interact through the cytosolic tail of CANX. The GST-TM-cytosolic Tail construct was transfected in Hela-CIITA cells and immunoprecipitated using anti-GST antibodies. Western blotting revealed that T6BP is co-immunoprecipitated with anti-GST in cells transfected with GST-TM-cytoTail and not in mock-transfected cells (Fig 7D). Taken together, we demonstrate here that T6BP interacts with CANX through its cytosolic tail. We propose that, through the binding to the cytosolic tail of*

CANX, T6BP promotes the interaction of CANX with CD74 leading to CD74 stabilization." We propose that through the binding to the cytosolic tail of CANX, T6BP promotes the interaction of CANX with CD74 leading to CD74 stabilization.

3. Although the HeLa-CIITA cells are a good model system, it would be important to confirm the role of TAX1BP1 for endogenous antigen presentation by MHC class II molecules in a professional antigen presenting cell. Since the authors already tried to assess monocyte-derived dendritic cells, but elicited their maturation during the investigated manipulations, B cell lines could be tested for dependence on TAX1BP1 for intracellular antigen presentation on MHC class II molecules.

As suggested by Reviewer-2, we intended to ask whether TAX1BP1 might affect antigen presentation in B cells. However, we could not get a reliable transfection of siRNA in B cells. In addition, due to the development/existence of compensatory/redundant mechanisms when knocking-down single autophagy receptor (please see our response to point-3 of Reviewer-1), we did not intend to use shRNA silencing in B cells. Thereafter, we decided to take an indirect approach and we now demonstrate that TAX1BP1 interacts with CANX in professional antigen presenting cells, namely B cells and dendritic cells. The following paragraph has been added page 14 and (Fig 7A-C): "*In HeLa-CIITA cells, we further confirmed the specific interaction between T6BP and CANX using two different techniques: 1) by immunoprecipitation of endogenous T6BP and endogenous CANX, we revealed that the IP fractions of anti-T6BP and anti-CANX IPs contained CANX and T6BP, respectively (Fig 7A-B); 2) by proximity ligation assay (PLA) on control cells and siT6BP-transfected cells a specific T6BP/CANX interaction was detected only in cells expressing T6BP (Fig 7C). To demonstrate that T6BP/CANX interaction is not restricted to Hela-CIITA cells, we then immunoprecipitated endogenous T6PB and CANX from two professional APCs, namely B cells and DC. As in Hela-CIITA, we revealed in the anti-T6BP and anti-CANX IP fractions, of both B cells and DC, the presence of CANX and T6BP, respectively (Fig 7A-B).*" Taken together, these results demonstrate that CANX interacts with TAX1BP1 directly or as part of a larger protein complex in immune cells, thus strongly suggesting that the role of TAX1BP1 in antigen presentation is not limited to non-professional APC.

Minor comments:

1.The text should refer to CD4+ T cells always in the same way. Currently, CD4 T cells, CD4superscript+ T cells and CD4+ T cells are in use.

Thank you for the comment. CD4superscript+ is now used throughout the article.

2.The authors describe the GFP moiety of their GFP-TAX1BP1 protein that they use for their immunoprecipitations as bait, but it would probably be more correct to describe the GFP as tag and TAX1BP1 as bait.

Also modified accordingly.

Reviewer #3:

We thank the reviewer-3 for highlighting that our study reports the novel discovery that T6BP1 plays a role in the selection of the peptide repertoire presented by MHC II.

We propose that TAX1BP1 acts at least at two distinct levels on MHC-II loading: 1) the positioning of late endosomes including the MIIC, thus influencing MHC-II molecule maturation and antigen degradation and 2) the function of CANX as a key component of the MHC-II loading complexe in the ER.

1) In this revised version, we show that the repositioning of the MIIC leads to the generation of unstable MHC αβ heterodimers which is most likely a direct consequence of CD74 exacerbated degradation. Importantly, we provide evidences that CD74 expression can be recovered when treating cells with chloroquine (Fig5D) and other cathepsin inhibitors (not shown). How T6BP dictates the trafficking of the MIIC remains to be determined but might be reminiscent of its action on endosomal trafficking.
2) In the present manuscript, we decide to focus on CANX that we identified as a protein partner of T6BP in our interactome because 1) it has been shown that CANX stabilizes CD74, in the ER, and thus favours the formation of CD74-MHC complexes. 2) we show that silencing of CANX recapitulates the observations made with T6BP-silenced cells. As suggested by the reviewers, to strengthen this point, we now provide insights in the molecular interactions between TAX1BP1 and CANX and new results supporting the physiological relevance of our findings. Using biochemical (co-IP) and proximity ligation assays, we now provide strong evidence demonstrating that TAX1BP1 interacts with Calnexin. We show that TAX1BP1 interacts with CANX in non-professional and in professional APC such as B cells and dendritic cells. Finally, using IP, we show that TAX1BP1 binds the cytosolic tail of CANX, which is known to regulate CANX functions. We propose that through the binding to the cytosolic tail of CANX, T6BP promotes the interaction of CANX with CD74 leading to CD74 stabilization.

….. the authors focused on calnexin because of a previously reported action of calnexin in degrading CD74. This is a justifiable choice, even if calnexin was among many candidates, the majority of which may not be reliable T6BP1 interacting partners. In this regard, it would have been very helpful to co-stain cells for calnexin and T6BP1, or calnexin and CD74, to increase confidence in direct interactions.

We thank reviewer-3 for highlighting that focusing on CANX is a justifiable choice and for the important suggestions. As suggested, we investigated further the link between TAX1BP1 and CANX. This is now highlighted page 14 and (Fig 7A-C): "*In HeLa-CIITA cells, we further confirmed the specific interaction between T6BP and CANX using two different techniques: 1) by immunoprecipitation of endogenous T6BP and endogenous CANX, we revealed that the IP fractions of anti-T6BP and anti-CANX IPs contained CANX and T6BP, respectively (Fig 7A-B); 2) by proximity ligation assay (PLA) on control cells and siT6BP-transfected cells a specific T6BP/CANX interaction was detected only in cells expressing T6BP (Fig 7C). To demonstrate that T6BP/CANX interaction is not restricted to Hela-CIITA cells, we then immunoprecipitated endogenous T6PB and CANX from two professional APCs, namely B cells and DC. As in Hela-CIITA, we revealed in the anti-T6BP and anti-CANX IP fractions, of both B cells and DC, the presence of CANX and T6BP, respectively (Fig 7A-B).*" Taken together, these results strongly support that CANX interacts with T6BP directly or as part of a larger protein complex.

…..The authors would do well to strengthen the proposed link between T6BP1/Calnexin/CD74 by performing additional experiments to show a specific and meaningful interaction between T6BP1 and calnexin.

To answer this specific point, we characterized on CANX the domain of interaction with TAX1BP1, these results are described page 15 and in (Fig 7F): "*In order to characterize the molecular interactions between CANX and T6BP, we then engineered a construct containing solely the transmembrane domain and the cytosolic tail of CANX fused to a GST tag. Since T6BP expression is mainly cytosolic, we reasoned that T6BP and CANX might interact through the cytosolic tail of CANX. The GST-TM-cytosolic Tail construct was transfected in Hela-CIITA cells and immunoprecipitated using anti-GST antibodies. Western blotting revealed that T6BP is co-immunoprecipitated with anti-GST in cells transfected with GST-TM-cytoTail and not in mock-transfected cells (Fig 7D). Taken together, we demonstrate here that T6BP interacts with CANX through its cytosolic tail.*"

This could be particularly pertinent to demonstrate that the absence of T6BP1 could decrease calnexin colocalization with CD74, which would explain increased degradation of the invariant chain when T6BP1 is silenced. We propose that through the binding to the cytosolic tail of CANX, T6BP promotes the interaction of CANX with CD74 leading to CD74 stabilization. However, since CD74 is dramatically and rapidly degraded in cells silenced for T6BP expression, looking for a CD74 colocalization with CANX in these cells is challenging.

Overall, the study remains primarily descriptive, with no insight into how T6BP1 affects the selection of the immunopeptidome (is this an indirect effect, given that T6BP1 does not seem to localize with the MIIC?).

The selection of the immunopeptidome is probably affected by:

a) the source protein of MHC-II ligands that is slightly different when T6BP in not expressed. To highlight this point, the following paragraph has been added page 9 and the results are presented in figure S2B: "*We then asked whether T6BP expression might affect the source of peptides, meaning the set of protein supplying peptide for MHC-II loading. To this end, we submitted the LC-MS/MS data to cell component enrichment analysis using Funrich software (Pathan et al, 2015). As expected from previous work (Dengjel et al., 2005; Marcu et al, 2021), according to Funrich annotation and as compared with the human proteome, in both siCTRL and siT6BP treated cells, the MHC-II-ligand source proteins were enriched in cellular fractions belonging to membranes, secretory pathways (e.g. exosomes and vesicles) but also to the cytosolic compartment of the cell (Fig S2B). In siCTRL treated cells MHC-II source proteins were also augmented in nuclear fractions, which was not the case for siT6BP-transfected cells. In contrast, MHC-II-ligand source proteins belonging to cytoplasma were enriched in siT6BP-treated cells (Fig S2B). Overall, this analysis shows that the bulk of MHC-II-ligand source proteins is provided by the same cellular fractions mainly membranes, secretory pathways and the cytosol. However, the presence of T6BP might also favour the presentation of peptide derived from the nucleus.*"

However, we believe that these differences in the source proteins cannot by themselves explain the influence of T6BP on MHC-II-restricted antigen presentation. A sentence has been added page 17 to highlight our point of view: *"However, although we observed, using functional enrichment analysis (Funrich, Fig S2), a slight influence of T6BP silencing on the cellular distribution of the source protein of MHC-II ligands, this can not by itself explain the effect of T6BP on the peptide repertoire, as T6BP expression also dictates the relative affinity of epitopes presented by MHC-II molecules."*

b) the activity of pH-sensitive cathepsines that are involved in antigen processing and CD74 cleavages. Indeed Accumulating evidence suggest that the positioning of intracellular vesicles controls their functions (Neefjes et al, 2017) and in particular the intravesicular pH (Johnson et al, 2016). To answer this point, we assessed the activity of cathepsin B/L in total cell extract of HeLa-CIITA cells transfected with siCRTL or siT6BP. In cells silenced for T6BP, we noticed a slight reduction/delay of cathepsin B/L activities. We decided not to include these data in the ms since they also do not provide a direct molecular link between T6BP and the selection of the immunopeptidome.

A direct link between T6BP1 and calnexin is tenuous, and the effect of calnexin on CD74 degradation had previously been described, leaving the proposed link from T6BP to CD74 and peptide loading neither certain nor entirely novel. The key would be to strengthen data on the interaction between T6BP1 and CD74, since this is the authors decided to focus on.

As mentioned above, we now provide a thorough demonstration that T6BP and CD74 interact in non-professional and professional APC. In particular, we demonstrate that T6BP binds the cytosolic tail of CANX that is known to regulate the various functions of CANX in the ER. Concerning the novelty of the findings, CANX / CD74 interactions have been studied using pulse chase experiments (Anderson & Cresswell, 1994), ectopic expression of mutant forms of CD74 (Romagnoli & Germain, 1995), Tunicamycin and endoglycosidase H treatments (Arunachalam & Cresswell, 1995) to perturb ER functions and study the role of glycans, respectively (Arunachalam & Cresswell, 1995). Expect if we missed some of the literature, we believe that we provide here the first direct demonstration that the lack of CANX expression (using siRNA targeting CANX) has a direct influence on the fate of CD74 leading to its degradation and thus on T cell activation.

1) In Fig1B, T cell activation seems quite modest in response to gag transfection, a positive control for maximal stimulation would have been helpful.

In these experiments, we use human primary CD4+ T cell clones specific for viral antigens (HIV gag and HCMH pp65). These cells are not immortalized but amplified upon *in vitro* restimulation using irradiated APC loaded with the cognate peptides and feeders. In particular, gag-specific CD4+ T cell clones have been extensively studied in their capacity to recognize HIV-loaded (Moris et al Blood 2006, Blanchet et al Immunnity 2010, Rodriguez-Plata et al JI 2012), -infected cells or Gag-transfected cells (Coulon et al JI 2016). Using ICS, we have shown that these gag-specific CD4+ T cells are capable of secreting a variety of cytokines upon peptide and whole-antigen stimulation (Almeida et al Blood 2009 & Coulon et al JI 2016). However, in contrast to IFNγ-Elispot, ICS requires a large number of T cells, that is the reason why we used IFNγ-Elispot for the siRNA and antigen screens (Fig 1). Others and we observed that in IFNγ-Elispot assays using human T cell clones, a maximum of one IFNγ+ spots can be detected for 10 to 50 clones, seeded in one well of an IFNγ-Elispot plates,

thus explaining the "relatively" low number of spots (Figure 1A). To circumvent this limitation, experiments are routinely performed in technical triplicates using 1000 and 5000 T cell clones per well. As positive control, we used APC loaded with the cognate peptide (Figure 1D and 1E). To answer to the reviewer-3's comments, the numbers of cell seeded per well are now indicated in the figure legend.
The normalization of the data in the right panel should be described - were the absolute counts between experiments very divergent or could they be combined without normalizing? Due to the nature of this primary T cell clones, the IFNγ-Elispot counts can be very divergent from one experiment to another that is why normalization is required to apply the Wilcoxon's test. The data normalization is now described in the Figure legend. In addition, as suggested by the reviwer-3, for clarity, we know indicated in the figure legends the range of T cell activation levels that we observed for the control (siCTRL) and siT6BP conditions in all panels of Figure-1.
The symbols for statistics should be better described - what comparisons to these refer to (vs. CTRL?)
We thank the reviewer for this helpful comment and we now provided a more detailed description of the symbols for statistics in the Figure legend.

2) In Fig1D, the authors should show absolute counts to show how the different Gag versions transfected compared with each other in mediating activation of T cells. The way data are normalized in the current figure do not allow the reader to determine if targeting Gag to the autophagy pathway vs. preventing this has a significant effect on its presentation and on T cell activation.
We thank the reviewer for this comment. We also believe that understanding the role of autophagy in antigen presentation is of upmost interest. In a previous study, we indeed asked whether targeting Gag to the autophagy pathway (using the fusion with LC3) has an influence on the capacity of APC to stimulate CD4+ T (Coulon et al JI 2016). We demonstrated that, in DC and HeLa-CIITA, specific targeting of gag to autophagosomes using LC3 fusion effectively enhances and broadens HIV-specific CD4+ T cell responses, thus favouring an endogenous MHC-II–restricted presentation (Coulon et al JI 2016). In this work, we confirmed that cell expressing Gag-LC3 have a better capacity to induce T cell activation than cell expressing Gag-LC3G120A that does not allow targeting of the fusion construct to autophagosomes. However, in this work the experiments were not designed to address this point, e.g the experiments with the 3 antigens were not systematically performed side by side. Our primary objective was to ask whether silencing T6BP might affect various endogenous antigen presentation pathways. So the comparison is pairwise siCTRL versus siT6BP for each antigen. This is now stated in the figure legend together with the absolute counts. The result section, page 6, has also been modified to further highlight the scientific question addressed here.
"*We next analysed the effect of T6BP silencing on autophagy-independent endogenous viral antigen processing by MHC-II molecules. As previously, HeLa-CIITA cells were first transfected with CTRL- or T6BP-targeting siRNAs (siCTRL and siT6BP respectively), then transfected with various plasmids encoding Gag, Gag-LC3$_{G120A}$ or Gag-LC3, and co-cultured with the Gag-specific CD4^{+} T cells (Fig 1D). Importantly, in our previous work we demonstrated that newly synthetized Gag is processed in an autophagy independent manner both in monocyte-derived DCs (MDDCs) and in HeLa-CIITA cells (Coulon et al., 2016). This was shown by using productively infected MDDCs and Gag-cDNA-transfected HeLa-CIITA cells treated with various drugs influencing autophagy (e.g. 3-MA, Spautin-1 and Torin-1), shRNA targeting LC3 or overexpressing a trans-dominant mutant of Atg4B (Atg4BC74A), which blocks the formation of autophagosomes. Using the same tools, we also showed that the fusion with LC3 (Gag-LC3) targets Gag to autophagosome-mediated degradation leading to an enhancement of CD4^{+} T cell activation. Gag-LC3$_{G120A}$ was also used as a negative control for autophagy-dependent degradation, as the G120A mutation in the C-terminus of LC3 abolishes the lipidation and incorporation of LC3 in the nascent membranes of autophagosomes, thus preventing Gag targeting into autophagosomes. Finally, we showed that upon Gag-LC3$_{G120A}$-transfection, Gag antigens are processed in an autophagy independent manner (Coulon et al., 2016).*"

3) In Fig2, the mock transfections serve very little purpose as currently analyzed. It would have been much more informative to have two separate siCTRL and two siT6BP1 measurements to compare within and across conditions (is variability between siCTRL and siT6BP1 larger than within either condition?). At a minimum, the authors should show how the siCTRL and siT6BP1 each compare to MOCK1 and MOCK2. We thank reviewer-3 for this important suggestion. In fact, in the field of immunopeptidomic the intrinsic variation of the method is usual addressed at the level of the instrumental (LC-MS/MS) variability by performing technical replicates. In our case for each condition, we used 5 technical replicates. However the variability at the level of the cells used to isolate the MHC-ligand is often unappreciated. Using different settings, Partridge T. et al. (Frontiers In Immunology 2018) and Alvaro-Benito M. et al. (Frontiers In Immunology 2018) recently underlined the variability of biological samples but nevertheless concluded that MHC-IP combined with LC-MS/MS is a qualitatively reproducible method. Nonetheless, we decided to take this parameter into account in our work and as suggested by the reviewer-3, we compared siRNA treated cells with

untreated cells. For each sample the % of MHC-II peptide ligand shared with the 3 other experimental conditions was determined and the mean % of shared MHC-II peptide ligand calculated. We observed that siT6BP treated cells shared significantly less MHC-II peptide ligands with the three other experimental conditions (Mock1, Mock2 or siCTRL). On the other hand, the mean % of shared peptide between mock treat cells (Mock1 or Mock2) and the cells transfected with the control (siCTRL) siRNA, were not significantly different. These data are now presented in Figure S2 and highlighted in the result section "*Overall, siT6BP treated cells shared significantly less MHC-II peptide ligands with the three other experimental conditions (Mock1, Mock2 or siCTRL) (Fig S2A). Together these results show that, although there is an intrinsic variability of the MHC-II ligandome in cells, the absence of T6BP has a pronounced and dramatic influence on the repertoire of peptides presented by MHC-II molecules.*"

4) Fig7A, the authors extrapolate from the strength of GFP vs. GFP-T6BP1 blotting that there was more GFP than fusion protein in the pull-downs, and then extrapolate that calnexin was 100-fold increased in the fusion IP. This is making the assumption that the GFP antibody binds equally well to GFP as to the fusion protein, which is not necessarily true. The calnexin blot shows very clear presence of the protein in the GFP-only IP. These data should be interpreted with more caution.
We thank reviewer-3 for this comment. The sentence has been removed from the results section. As mentioned above, page 14 and (Fig 7A-C), we now focus the point on the co-IP of endogenous T6BP and endogenous CANX.

Reviewer #3 (Significance (Required)):

We also believe that "this study provides a new avenue for further exploration". We focused here on the role of CANX / T6BP interactions as one pathway by which T6BP has an impact on MHC II antigen presentation. We now provide strong evidence that T6BP interacts with CANX 1) in professional APC such as B cells and DC; 2) through the cytosolic tail of CANX whose post-translational modifications are known to regulate its various ER functions.

Referees cross-commenting

I largely concur with comments made by reviewer's 1 and 2.
I do think that the use of siRNA is adequate - if sufficiently controlled, and that the authors would not need to repeat their studies with CRISPR KO cells at this point. In this respect, reviewer 1 makes a useful suggestion to use an siRNA insensitive transgene to show that the knockdown phenotype can be reversed by 'rescue experiments'. That said, I do not see this as a critical weakness. Rather, the lack of mechanistic insight, and - as pointed out by the two other reviewers - the fact that the authors used only a single model cell line (HeLa CIITA) in their study are the key points that should be addressed to increase significance.

Dear Dr. Moris,

Thank you for transferring your revised manuscript from Review Commons to EMBO reports. I have now received the reports from the three referees that had assessed your study at RC that were asked to re-evaluate the manuscript. Please find their comments below. As you will see, referee #1 (RC referee #2) and referee #2 (RC referee #3) now fully support publication of your study in EMBO reports. Referee #3 (RC referee #1) has some remaining concerns and further suggestions to improve the manuscript, I ask you to address in a final revised manuscript.

Moreover, the manuscript needs formatting according to our journal style. Please carefully review the instructions that follow below.

PLEASE NOTE THAT upon resubmission revised manuscripts are subjected to an initial quality control prior to exposition to re-review. Upon failure in the initial quality control, the manuscripts are sent back to the authors, which may lead to delays. Frequent reasons for such a failure are the lack of the data availability section (please see below) and the presence of statistics based on n=2 (the authors are then asked to present scatter plots or provide more data points).

When submitting your final revised manuscript, we will require:

1) a .docx formatted version of the final manuscript text (including legends for main figures, EV figures and tables), but without the figures included. Figure legends should be compiled at the end of the manuscript text.

2) individual production quality figure files as .eps, .tif, .jpg (one file per figure), of main figures and EV figures. Please upload these as separate, individual files upon re-submission.

The Expanded View format, which will be displayed in the main HTML of the paper in a collapsible format, has replaced the Supplementary information. You can submit up to 5 images as Expanded View. Please follow the nomenclature Figure EV1, Figure EV2 etc. The figure legend for these should be included in the main manuscript document file in a section called Expanded View Figure Legends after the main Figure Legends section. Additional Supplementary material should be supplied as a single pdf file labeled Appendix. The Appendix should have page numbers and needs to include a table of content on the first page (with page numbers) and legends for all content. Please follow the nomenclature Appendix Figure Sx, Appendix Table Sx etc. throughout the text, and also label the figures and tables according to this nomenclature.

For more details, please refer to our guide to authors:
http://www.embopress.org/page/journal/14693178/authorguide#manuscriptpreparation

Please consult our guide for figure preparation:
http://wol-prod-cdn.literatumonline.com/pb-assets/embo-site/EMBOPress_Figure_Guidelines_061115-1561436025777.pdf

See also the guidelines for figure legend preparation:
https://www.embopress.org/page/journal/14693178/authorguide#figureformat

3) a complete author checklist, which you can download from our author guidelines (https://www.embopress.org/page/journal/14693178/authorguide). Please insert page numbers in the checklist to indicate where the requested information can be found in the manuscript. The completed author checklist will also be part of the RPF.

Please also follow our guidelines for the use of living organisms, and the respective reporting guidelines:
http://www.embopress.org/page/journal/14693178/authorguide#livingorganisms

4) that primary datasets produced in this study (e.g. RNA-seq, ChIP-seq, structural and array data) are deposited in an appropriate public database. If no primary datasets have been deposited, please also state this in a dedicated section (e.g. 'No primary datasets have been generated and deposited'), see below.

See also: http://embor.embopress.org/authorguide#datadeposition

Please remember to provide a reviewer password if the datasets are not yet public.

The accession numbers and database should be listed in a formal "Data Availability" section (placed after Materials & Methods) that follows the model below. This is now mandatory (like the COI statement). Please note that the Data Availability Section is restricted to new primary data that are part of this study. This section is mandatory. As indicated above, if no primary datasets have been deposited, please state this in this section

Data availability

The datasets produced in this study are available in the following databases:

- RNA-Seq data: Gene Expression Omnibus GSE46843 (https://www.ncbi.nlm.nih.gov/geo/query/acc.cgi?acc=GSE46843)
- [data type]: [name of the resource] [accession number/identifier/doi] ([URL or identifiers.org/DATABASE:ACCESSION])

*** Note - All links should resolve to a page where the data can be accessed. ***

Moreover, I have these editorial requests:

6) As they are significantly cropped, please provide the source data for the Western blots shown in the manuscript (including the EV figures). The source data will be published in separate source data files online along with the accepted manuscript and will be linked to the relevant figures. Please submit scans of entire gels or blots together with the final revised manuscript. Please include size markers for scans of entire gels, label the scans with figure and panel number, and send one PDF file per figure (main and EV figures).

7) Our journal encourages inclusion of *data citations in the reference list* to directly cite datasets that were re-used and obtained from public databases. Data citations in the article text are distinct from normal bibliographical citations and should directly link to the database records from which the data can be accessed. In the main text, data citations are formatted as follows: "Data ref: Smith et al, 2001" or "Data ref: NCBI Sequence Read Archive PRJNA342805, 2017". In the Reference list, data citations must be labeled with "[DATASET]". A data reference must provide the database name, accession number/identifiers and a resolvable link to the landing page from which the data can be accessed at the end of the reference. Further instructions are available at: http://www.embopress.org/page/journal/14693178/authorguide#referencesformat

8) Regarding data quantification and statistics, please make sure that the number "n" for how many independent experiments were performed, their nature (biological versus technical replicates), the bars and error bars (e.g. SEM, SD) and the test used to calculate p-values is indicated in the respective figure legends (also for potential EV figures and all those in the final Appendix). Please also check that all the p-values are explained in the legend, and that these fit to those shown in the figure. Please provide statistical testing where applicable. Please add complete statistical testing to all diagrams (for main, EV figures). Please also indicate (e.g. with n.s.) if testing was performed, but the differences are not significant. Please avoid the phrase 'independent experiment', but clearly state if these were biological or technical replicates. See also: http://www.embopress.org/page/journal/14693178/authorguide#statisticalanalysis

9) Please also note our reference format:
http://www.embopress.org/page/journal/14693178/authorguide#referencesformat

10) For microscopic images, please add scale bars of similar style and thickness to all the microscopic images, using clearly visible black or white bars (depending on the background). Please place these in the lower right corner of the images. Please do not write on or near the bars in the image but define the size in the respective figure legend.

11) We updated our journal's competing interests policy in January 2022 and request authors to consider both actual and perceived competing interests. Please review the policy https://www.embopress.org/competing-interests and update your competing interests if necessary. Please name this section 'Disclosure and Competing Interests Statement' and put it after the Acknowledgements section.

12) Please make sure that all the funding information is entered into the online submission system and is complete and similar to the one in the acknowledgements section of the manuscript text file.

13) Please provide a more comprehensive title with not more than 100 characters (including spaces). Please do not use the word 'novel' in the title. EMBO reports always tries to publish novel insight. See also the comment by referee #1.

14) Please name the primer table either Table 1 (and move it with a legend to the end of the manuscript text file) or include this in the Appendix file (as Appendix Table S1), provided there will be an Appendix.

15) Table S1 seems to be a dataset. Please name this Dataset EV1 and upload it as dataset file (excel file) with a legend on the first TAB. Finally, please change the callouts for this item to Dataset EV1.

16) Please make sure that the manuscript sections are ordered like this:
Title page - Abstract - Introduction - Results - Discussion - Materials and Methods - Data availability section (DAS) - Acknowledgements - Author contributions - Disclosure and Competing Interests Statement - References - Figure legends - Expanded View Figure legends

In addition, I would need from you:
- a short, two-sentence summary of the manuscript (not more than 35 words).

- three to four short one sentence bullet points highlighting the key findings of your study.
- a schematic summary figure (synopsis image) in jpeg or tiff format with the exact width of 550 pixels and a height of not more than 400 pixels that can be used as a visual synopsis on our website.

I look forward to seeing a revised version of your manuscript when it is ready. Please let me know if you have questions or comments regarding the revision.

Please use this link to submit your revision: https://embor.msubmit.net/cgi-bin/main.plex

Yours sincerely,

Achim Breiling
Senior Editor
EMBO Reports

Referee #1:

The authors demonstrate that among several autophagy receptors (p62, NDP52, optineurin and TAX1BP1) only TAX1BP1 silencing decreases intracellular antigen presentation by MHC class II molecules of HeLa-CIITA cells. This affects both antigens that are targeted to autophagosomes via fusion with LC3 and untargeted antigens, but not extracellular peptide presentation by MHC class II molecules. Accordingly, the MHC class II, but not the MHC class I ligandome is changed upon TAX1BP1 silencing. While the location of the MHC class II containing compartment (MIIC) is altered in the absence of TAX1BP1 MHC class II internalization is not affected. Instead, invariant chain (Ii)/CD74 degradation is increased upon TAX1BP1 loss. The authors identify calnexin as an interaction partner of TAX1BP1 and calnexin silencing phenocopies TAX1BP1 loss. From these findings the authors conclude that TAX1BP1 stabilizes CD74 for efficient complex formation with MHC class II molecules, and increased CD74 degradation in the absence of TAX1BP1 compromises MHC class II presentation of intracellular antigens.

In their revised manuscript version (now submitted to EMBO Reports) the authors have addressed all of my previous concerns. They characterized the altered MHC class II ligandome upon TAX1BP1 silencing with respect to protein source and report interesting findings with respect to possible decreased nuclear antigen, but increased mitochondrial antigen presentation. Furthermore, they characterize the cytosolic calnexin domain as TAX1BP1 interactor. Finally, they demonstrate TAX1BP1 interaction with calnexin also in B and dendritic cells. These new experimental data significantly strengthen the manuscript and strongly suggest that TAX1BP1 interaction with calnexin facilitates calnexin mediated stabilization of Ii for more efficient MHC class II loading.

Minor comments:
1. Currently the manuscript title is not very informative. The authors might consider changing it to convey the main message of their study. Along these lines, "The autophagy receptor TAX1BP1 (T6BP) improves antigen presentation by MHC class II molecules" or "The autophagy receptor TAX1BP1 (T6BP) stabilizes CD74 for improved MHC class II restricted antigen presentation" could be considered.

Referee #2:

The authors have provided additional data and edited the manuscript in response to my comments. They have largely addressed all of the points that I raised in my initial review and I find the manuscript much improved. I have no further comments.

Referee #3:

In this version of the manuscript, authors provided a good mechanistic insight into how TAX1BP1 may be involved in the antigen presentation process by further questioning MHC-II circulation/loading and the proposed link between TAX1BP1, CANX, and CD74 by using additional controls in the existing data and performing additional IP experiments. All the experimental and explanatory efforts to answer the concerns that were made by the all the reviewers are well appreciated. In my opinion, the revised version of the manuscript is now more suitable for publication; however, authors still need to clarify some additional critical points made below.

1. Do the authors see any effect of TAX1BP1 overexpression on antigen presentation? Please describe in the manuscript.

2. In mechanistic studies, rescue experiments are key to reveal the true nature of proposed links between the proteins in action.

The authors pointed that triple transfection of the cells might affect their viability and so on. This point could be tackled by producing stable expression cell lines or using inducible expression plasmids. I think that proposing a link between TAX1BP1 and antigen presentation is valuable and should be placed on a more solid ground as it is the key element in the manuscript (e.g., overexpression and rescue experiments may be performed)

3. The authors are proposing that "...T6BP silencing affects CANX functions resulting in CD74 degradation and aberrant MHC-II peptide loading and antigen presentation to CD4+ T cells" which is another piece of data that requires corroboration via rescue experiments. How does rescue of CD74 degradation affect MHC-II peptide loading and antigen presentation? This point has got rather short shrift and deserves a better explanation to strengthen the proposed link between T6BP1, Calnexin and CD74. I suggest authors move figure 6 (which may not be critical to show as a main figure) to supplementary and elaborate more on the proposed link which is one of the main interests of the manuscript in a new figure, or by extending the figure 7.

4. In figure S7D, it has been shown that "3-fold enrichment of CANX in cells transfected with GFP-T6BP as compared to GFP-transfected cells" by an IP experiment. First, all IP experiments require input controls with immunoblotting rather than Coomassie staining. In addition, how do the authors explain that GFP-T6BP increased CANX interaction in S7D but paradoxically, in figure S7E, siRNA T6BP increased the expression of CANX? On the other hand, it has been shown that siRNA CANX recapitulates siRNA T6BP in terms of activating T cells. Hence a link proposed between these two proteins, in terms of antigen presentation; however, considering that siRNA T6BP increasing expression of CANX in fig S7E, the observed effects of these proteins might be independent from each other, or siRNA T6BP might have some effects on cellular localization/trafficking of CANX. This point needs to be clarified. The CANX and Actin blots in figure 7E needs to be replaced with a better one.

5. Even though authors showed the interaction between CANX and T6BP, and that silencing of these proteins separately reduces T-cell activation (Fig 7), this does not prove the point that their interaction is required for antigen presentation. Or do they have independent effects? This is an important question to answer in terms of proposing a potential mechanism for the role of the T6BP1/Calnexin/CD74 link on antigen presentation.

6. In figure 7D, instead of mock treatment, GST overexpression should be used to confirm TM-CytoTail-CANX specific pulldown.

7. The IP experiment in fig 7A and 7B requires additional controls to exclude non-specific binding to the beads (e.g., silencing the bait in the figures or involving IgG coupled beads as a control).

8. It would be better to add the siCANX result in figure 7C for further confirmation of the proposed link.

9. Present error bars and statistical analysis for the all the graphs in figure 5. In addition, activity of the CQ and Epo treatments should be checked. Even though they are well-known inhibitors, authors are supposed to show that they worked in their hands as well.

10. In figure 5D, CQ treatment looks like increasing CD74 level in the CTRL conditions. Please comment on this point and question whether it is an autophagy target or not.

11. Please show the IP results and controls for Fig S3A.

We would like to thank referee#3 for helpful comments and suggestions that have clearly improved the quality of the manuscript. We also strongly appreciate that the referee#3 acknowledges our effort to answer the reviewer's concerns and that our work now reaches standards for publication.

1. Do the authors see any effect of TAX1BP1 overexpression on antigen presentation? Please describe in the manuscript. 2. In mechanistic studies, rescue experiments are key to reveal the true nature of proposed links between the proteins in action. The authors pointed that triple transfection of the cells might affect their viability and so on. This point could be tackled by producing stable expression cell lines or using inducible expression plasmids. I think that proposing a link between TAX1BP1 and antigen presentation is valuable and should be placed on a more solid ground as it is the key element in the manuscript (e.g., overexpression and rescue experiments may be performed).

We fully agree with referee#3 that proposing a link between TAX1BP1 and antigen presentation is valuable. Using multiple techniques including T-cell activation assays, immunopeptidomics, confocal imaging of the MHC-II loading compartments, we provide strong evidence that altering T6BP expression has a major influence on virus-specific CD4+ T-cell activation, on both the diversity and the quality of the MHC-II ligands and overall on MHC-II biology. We fully agree that rescue experiments might help in narrowing down the mechanism of action, using T6BP mutants for instance. However, due to the functional redundancy of autophagy receptor family members, others (Lazarou et al) and we experienced troubles in performing these rescue experiments. Our results were then validated in independent experiments using multiple siRNA from different providers.

Nonetheless, as requested by the referee, we performed an overexpression experiment. In brief, Hela-CIITA cells were co-tranfected with plasmids encoding T6BP-GFP and Gag antigen and co-cultured with Gag-specific CD4+ T cells. T cell activation was then monitored using IFNγ-Elispot. As controls, cells were transfected with GFP-encoding plasmid and/or loaded exogenously with the cognate peptide (Gag2). The data are presented in the figure below.

Overexpression of T6BP in Hela-CIITA cell seems to enhance CD4+ T cell activation (at the different APC/T cell ratio used). However, the effect might not be limited to cells expressing the antigen as Hela-CIITA transfected with T6BP-GFP and then exogenously loaded with the cognate peptide, also induced a higher T-cell activation as compared with cells expressing GFP. These results further confirm the role of T6BP in MHC-II biology and T cell activation. However, there is no straightforward explanation for this enhancement of T cell activation upon T6BP overexpression. One could speculate that the delivery of MHC-II molecules at the cell surface might be enhanced due to further stabilisation of CD74 and/or that the repertoire of peptide might be different allowing better binding of the peptide added exogenously etc. We believe that although interesting these observations do not provide further information on the mechanistic insight of T6BP involvement in MHC-II biology. Thereafter, the results are presented, as requested by Referee#3, but not included in the manuscript.

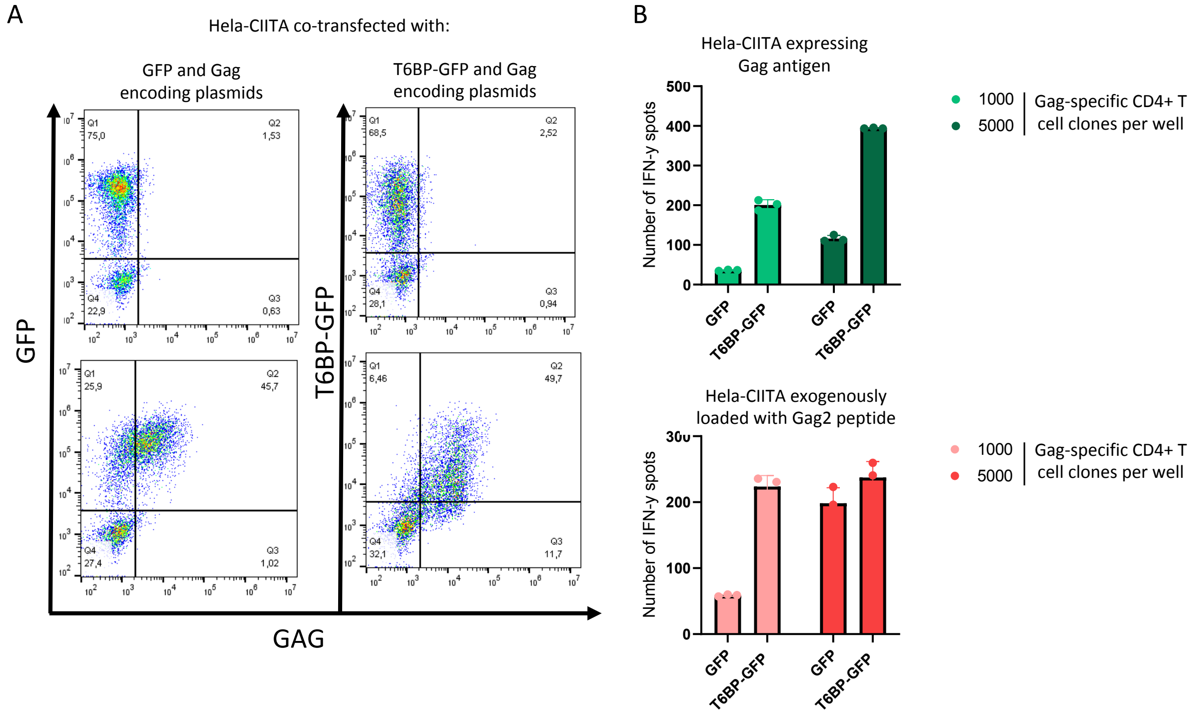

3. The authors are proposing that "...T6BP silencing affects CANX functions resulting in CD74 degradation and aberrant MHC-II peptide loading and antigen presentation to CD4+ T cells" which is another piece of data that requires corroboration via rescue experiments. How does rescue of CD74 degradation affect MHC-II peptide loading and antigen presentation? This point has got rather short shrift and deserves a better explanation to strengthen the proposed link between T6BP1, Calnexin and CD74. I suggest authors move figure 6 (which may not be critical to show as a main figure) to supplementary and elaborate more on the proposed link which is one of the main interests of the manuscript in a new figure, or by extending the figure 7.
We agree that rescuing CD74 expression would sustain our conclusions. We actually intended to perform rescue experiments and functional assays (assessing the influence on T cell activation). To this end, we rescued CD74 expression using various concentrations of drugs affecting acidification (such as chloroquine in Figure-5) or inhibiting cathepsins, involved in CD74 degradation. However, these experiments were not conclusive since the drug treatments also affected the presentation of viral antigens by wild type cells (naturally expressing CD74). In fact, this was to be expected since a fine-tuning of CD74 degradation is required, in the MHC-II loading compartments, to liberate the so-called CLIP peptide (derived from CD74) in the peptide-binding groove of the MHC-II molecule. This CLIP peptide then competes with and is replaced by high affinity antigenic peptides in the peptide-binding groove. Thereafter, any modulation of CD74 expression and degradation will ultimately affect the MHC-II peptide repertoire and CD4+ T cell activation. This has been exemplified by others using biochemical and peptidomic approaches (e.g. Muntasell A et al, JI 2004; Zhou Z et al, EJI 2017; Thompson JA et al, Cancer Immunol Immunother 2008). We believe that rescuing CD74 expression by cDNA transfection will lead to similar observations and conclusions and will not allow strengthening the proposed link between T6BP, Calnexin and CD74.

4**. In figure S7D, it has been shown that "3-fold enrichment of CANX in cells transfected with GFP-T6BP as compared to GFP-transfected cells" by an IP experiment. First, all IP experiments require input controls with immunoblotting rather than Coomassie staining.** Thanks for these remarks. The input for the CANX IP has been added in FigS7D (now called Appendix Figures-S3D). The input control (lysat) of the GFP IP experiments are already presented in FigS7B (now called Appendix Figures-S3B). The Coomassie staining is shown as a quality control for the materiel used in LC-MS/MS experiments. Overall, these experiments confirm that T6BP interacts with CANX.

In addition, how do the authors explain that GFP-T6BP increased CANX interaction in S7D but paradoxically, in figure S7E, siRNA T6BP increased the expression of CANX? We apologize that the quality of the WB shown in FigS7E (now called Appendix Figures-S3E) lead to a misunderstanding. Indeed, overall, we did not observe a significant increase of CANX expression in cells treated with siT6BP (see Fig7E for instance). In fact, in FigS7E (now called Appendix Figures-S3E) there is more material loaded in the lane corresponding to siT6BP as compared to siCRTL and siCANX, please see the actin blot. To clarify this point we now quantify the band intensities in FigS7E (now called Appendix Figures-S3E) and indeed there is no significant variation of CANX expression in cells treated with siT6BP.

On the other hand, it has been shown that siRNA CANX recapitulates siRNA T6BP in terms of activating T cells. Hence a link proposed between these two proteins, in terms of antigen presentation; however, considering that siRNA T6BP increasing expression of CANX in fig S7E, the observed effects of these proteins might be independent from each other, or siRNA T6BP might have some effects on cellular localization/trafficking of CANX. This point needs to be clarified. As mentioned above, we did not observed a variation of CANX expression in cells treated with siT6BP (see FigS7E (now called Appendix Figures-S3E) and Fig7E.

The CANX and Actin blots in figure 7E needs to be replaced with a better one: As requested, the CANX, T6BP and Actin blots were replaced by blots with better resolutions.

5. **Even though authors showed the interaction between CANX and T6BP, and that silencing of these proteins separately reduces T-cell activation (Fig 7), this does not prove the point that their interaction is required for antigen presentation. Or do they have independent effects? This is an important question to answer in terms of proposing a potential mechanism for the role of the T6BP1/Calnexin/CD74 link on antigen presentation.** We understand the point of referee#3 and we wish we had a straightforward answer to this question. However, we are again here blocked by the redundancy of autophagy receptors that prohibit using Crispr/cas KO. We are currently developing the tools, such as CANX KO cells, to address this point using CANX mutants in the future. Again, we might encounter difficulties due to the partial function overlap between CANX and Calreticulin, the other ER chaperone involved in MHC-biology, (see for instance Molinari et al,

Molecular Cell 2004 or Danilczyk et al, JBC 2000). Nonetheless, we believe that we provide here a rigorous biochemical and immunological demonstration that T6BP is involved in MHC-II biology and thus antigen presentation.

6. In figure 7D, instead of mock treatment, GST overexpression should be used to confirm TM-CytoTail-CANX specific pulldown. We agree with the referee, the wording "mock treated" is incorrect. In the text and Fig7D legend, we changed it to cells transfected with the "control vector".

7. The IP experiment in fig 7A and 7B requires additional controls to exclude non-specific binding to the beads (e.g., silencing the bait in the figures or involving IgG coupled beads as a control). The requested controls (IP procedures performed with beads not coupled to antibodies) have now been added to each panel. Overall the results highlight that T6BP and CANX interact in a specific manner.

8. It would be better to add the siCANX result in figure 7C for further confirmation of the proposed link. For the PLA experiments, the siT6BP is an internal control showing the specificity of the PLA co-staining. We believe that adding siCANX might be elegant but will not provide additional information.

9. Present error bars and statistical analysis for the all the graphs in figure 5. In addition, activity of the CQ and Epo treatments should be checked. Even though they are well-known inhibitors, authors are supposed to show that they worked in their hands as well.
We thank the reviewer for these suggestions. Error bars and statistical analysis were added to Figure 5D. In addition, the efficiency of Epoxomycin-mediated proteasomal inhibition was controlled by analysing the accumulation of polyubiquitinated proteins in HeLa-CIITA cells treated with Epoxomycin using the same batch, concentration and kinetic as in the main figure. These results are presented in Appendix Figure-S3 and highlighted in the results section page 22: "*In contrast, although proteasomal inhibition induced, as expected, the accumulation of polyubiquitinated proteins (Appendix Figure-S3), Epoxomycin treatment did not affect the expression levels of CD74 (Fig 5D).*"
Note that the functionality of the Choloroquine treatment in blocking endo-lysosomal acidification, is already illustrated in the Figure 5D with the expected accumulation of the Iip22 fragment of CD74.

10. **In figure 5D, CQ treatment looks like increasing CD74 level in the CTRL conditions. Please comment on this point and question whether it is an autophagy target or not.** As mentioned above, a fine-tuning of CD74 degradation is required, in the MHC-II loading compartments (MIIC), to liberate the CLIP peptide, derived from CD74, in the peptide-binding groove of the MHC-II molecule. This CLIP peptide then competes with and is replaced by high affinity antigenic peptides by the chaperone HLA-DM. In the MIIC, the degradation of CD74 is controlled by pH-sensitive cathepsins. Thereafter inhibition of acidification using Chloroquine is expected to inhibit cathepsin activity which results in the accumulation of full-length CD74 and the intermediate fragments such as p22, seen in Figure 5D. A sentence and references have been added page-12 in the result section to explain this point:
"As expected, since the proteolytic cleavages of CD74, required for MHC-II peptide-loading are dependent on cathepsin activities (Manoury et al., 2003; Nakagawa et al., 1998; Riese et al., 1996; Shi et al., 2000), the treatment of control cells with Chloroquine allowed the detection of the intermediate degradation fragment Iip22 (Fig 5D)."

11. Please show the IP results and controls for Fig S3A. To analyse the quality of the MHC-I immunoprecipation, we analyse the length distribution and biochemical properties (prediction to bind the HLA class I molecules expressed by the cells) of the peptide identified by LC-MS/MS. Thereafter, control gels to check for the quality of the MHCI IP are not routinely performed (see for instance Nelde et al, 2019 and Ghosh et al, 2019). The peptide presented in Figure S3A (now called Dataset EV2 (related to Figure-3) fulfil these criteria (on average 9 amino acid long and capacity to bind HLA class I alleles from HeLa-CIITA cells).

1st Revision - Editorial Decision 8th Sep 2022

Dear Dr. Moris,

Thank you for the submission of your revised manuscript to our editorial offices. I have now received the reports from the referee that I asked to re-evaluate your study, you will find below. As you will see, the referee now fully supports the publication of your study.

Before proceeding with formal acceptance, I have these editorial requests I ask you to address in a final revised manuscript.

- Please provide the abstract written in present tense throughout and with not more than 175 words.

- We now use CRediT to specify the contributions of each author in the journal submission system. CRediT replaces the author contribution section. Please use the free text box to provide more detailed descriptions. Thus, please remove the author contributions section from the manuscript text file. See also guide to authors: https://www.embopress.org/page/journal/14693178/authorguide#authorshipguidelines

- Per journal policy, we do not allow 'data not shown', which is stated several times in the manuscript. All data referred to in the paper should be displayed in the main or Expanded View figures, or an Appendix. Thus, please add these data (or change the text accordingly if these data are not central to the study). See: http://embor.embopress.org/authorguide#unpublisheddata

- In the "Data Availability section" (DAS) please add direct links to the two datasets and make sure these are public latest upon publication of the study.

- Please make sure that the number "n" for how many independent experiments were performed, their nature (biological versus technical replicates), the bars and error bars (e.g. SEM, SD) and the test used to calculate p-values is indicated in the respective figure legends (main and EV figures), and that statistical testing has been done where applicable. Please avoid phrases like 'independent experiment', but clearly state if these were biological or technical replicates. Please add complete statistical testing to all diagrams (main and EV figures). Please also indicate (e.g. with n.s.) if testing was performed, but the differences are not significant. In case n=2, please show the data as separate datapoints without error bars and statistics.

- Please add scale bars of similar style and thickness to all the microscopic images, using clearly visible black or white bars (depending on the background). Please place these in the lower right corner of the images themselves. Please do not write on or near the scale bars, but define the size in the legend. Presently, many scale bars are too small and most of them have numbers near them (that will be hard to see in the online versions of the figures).

- For Appendix Figure S4C, do you have a version without the crack (or whatever happened here to the gel/blot)? If not, please add a note to the legend explaining what has happened.

- Please make sure that all figure panels are called out, that they are called out separately and sequentially. Presently, Fig. 1C is called out before 1B, Fig. 2C is called out before 2B and there seems to be no callout for Fig. 3C. Please check.

- Finally, please find attached a word file of the manuscript text (provided by our publisher) with changes we ask you to include in your final manuscript text, and some queries, we ask you to address. Please provide your final manuscript file (using the attached file as basis) with track changes, in order that we can see any modifications done.

In addition, I would need from you:
- a short, two-sentence summary of the manuscript (not more than 35 words).
- two to four short (!) bullet points highlighting the key findings of your study (two lines each).
- a schematic summary figure (in jpeg, png or tiff format with the exact width of 550 pixels and a height of not more than 400 pixels) that can be used as a visual synopsis on our website.

I look forward to seeing the final revised version of your manuscript when it is ready. Please let me know if you have questions regarding the revision.

Please use this link to submit your revision: https://embor.msubmit.net/cgi-bin/main.plex

Best,

Achim Breiling
Senior Editor
EMBO Reports

Referee #3:

The authors have provided additional data and edited the manuscript in response to my comments. Even though they have answered most of my comments without experiments, they have covered experimental controls very well. I believe that the manuscript has shown great development since its first submission, and now has my consent for publication.

Dear Dr. Breiling,

We are grateful that the last referee now fully supports the publication of your study. Thank you for accepting our work for publication in EMBO Reports. It's a great news!

Please find the final revised version of our manuscript. We have addressed the editorial requests:

- the abstract written is now in present tense and with no more than 175 words.
- the author contributions section has been removed
- the quotation of the data not central to the study have been removed.
- In the DAS a direct link has been added. To make the two datasets public, we will need a DOI.
- Figure legends of the main and EV figures have been edited according to guidelines and answering to the questions in the word file of the manuscript text provided by your publisher.
- Scale bars of similar style and thickness have been added to all the confocal microscopic images, using white bars, at the lower right corner of the images themselves. For some of the merge images we could not easily remove the writing near the scale bars. We hope this will be ok.
- For Appendix Figure S4C, a note has been added in the figure legend.
- We checked the figure panels, all figure panels should be now called out and sequentially.
- Please also find:
    - the short, two-sentence summary of the manuscript:

"The autophagy receptor T6BP (TAX1BP1) stabiles the invariant chain (CD74) and regulates MHC-II molecule (MHC-II) trafficking, thereby favouring the presentation of high affinity peptides to virus-specific CD4+ T cells"

- four short one-sentence bullet points highlighting the key findings of our study:

• T6BP loss prevents the activation of HIV- and HCMV-specific CD4+ T cell clones.
• T6BP expression affects the quality and the diversity of peptides presented by MHC-II molecules
• T6BP interacts with the cytoplasmic tail of calnexin to favour MHC-II molecule chaperoning by the invariant cain (CD74)
• Calnexin loss replicates the functional consequences of T6BP silencing: decreased CD4+ T cell activation and exacerbated CD74 degradation.

- And also the schematic summary figure.

Best regards,

INSTITUT DE BIOLOGIE INTEGRATIVE DE LA CELLULE (I2BC)
Unité mixte CEA – CNRS – Université Paris-Saclay - UMR 9198
avenue de la terrasse, F-91190 Gif-sur-Yvette - Tél : 33 1 69 82 62 93 - courriel : arnaud.moris@i2bc.paris-saclay.fr

2nd Revision - Editorial Decision 23rd Sep 2022

Arnaud Moris
University Paris-Saclay, CEA, CNRS, Institute for Integrative Biology of the Cell (I2BC)
France

Dear Dr. Moris,

I am very pleased to accept your manuscript for publication in the next available issue of EMBO reports. Thank you for your contribution to our journal.

At the end of this email I include important information about how to proceed. Please ensure that you take the time to read the information and complete and return the necessary forms to allow us to publish your manuscript as quickly as possible.

As part of the EMBO publication's Transparent Editorial Process, EMBO reports publishes online a Review Process File to accompany accepted manuscripts. As you are aware, this File will be published in conjunction with your paper and will include the referee reports, your point-by-point response and all pertinent correspondence relating to the manuscript.

If you do NOT want this File to be published, please inform the editorial office within 2 days, if you have not done so already, otherwise the File will be published by default [contact: emboreports@embo.org]. If you do opt out, the Review Process File link will point to the following statement: "No Review Process File is available with this article, as the authors have chosen not to make the review process public in this case."

Thank you again for your contribution to EMBO reports and congratulations on a successful publication. Please consider us again in the future for your most exciting work.

Yours sincerely,

Achim Breiling
Editor
EMBO Reports

****

THINGS TO DO NOW:

Please note that you will be contacted by Wiley Author Services to complete licensing and payment information. The required 'Page Charges Authorization Form' is available here: https://www.embopress.org/pb-assets/embo-site/er_apc.pdf - please download and complete the form and return to embopressproduction@wiley.com

EMBO Press participates in many Publish and Read agreements that allow authors to publish Open Access with reduced/no publication charges. Check your eligibility: https://authorservices.wiley.com/author-resources/Journal-Authors/open-access/affiliation-policies-payments/index.html

You will receive proofs by e-mail approximately 2-3 weeks after all relevant files have been sent to our Production Office; you should return your corrections within 2 days of receiving the proofs.

Please inform us if there is likely to be any difficulty in reaching you at the above address at that time. Failure to meet our deadlines may result in a delay of publication, or publication without your corrections.

All further communications concerning your paper should quote reference number EMBOR-2022-55470V3 and be addressed to emboreports@wiley.com.

Should you be planning a Press Release on your article, please get in contact with emboreports@wiley.com as early as possible, in order to coordinate publication and release dates.

EMBO Press Author Checklist

Corresponding Author Name: Arnaud Moris	
Journal Submitted to: Embo Reports	
Manuscript Number: #EMBOR-2022-55470V1	

USEFUL LINKS FOR COMPLETING THIS FORM
The EMBO Journal - Author Guidelines
EMBO Reports - Author Guidelines
Molecular Systems Biology - Author Guidelines
EMBO Molecular Medicine - Author Guidelines

Reporting Checklist for Life Science Articles (updated January 2022)
This checklist is adapted from Materials Design Analysis Reporting (MDAR) Checklist for Authors. MDAR establishes a minimum set of requirements in transparent reporting in the life sciences (see Statement of Task: 10.31222/osf.io/9sm4x). Please follow the journal's guidelines in preparing your manuscript.
Please note that a copy of this checklist will be published alongside your article.

Abridged guidelines for figures
1. Data
The data shown in figures should satisfy the following conditions:
- the data were obtained and processed according to the field's best practice and are presented to reflect the results of the experiments in an accurate and unbiased manner.
- ideally, figure panels should include only measurements that are directly comparable to each other and obtained with the same assay.
- plots include clearly labeled error bars for independent experiments and sample sizes. Unless justified, error bars should not be shown for technical replicates.
- if n<5, the individual data points from each experiment should be plotted. Any statistical test employed should be justified.
- Source Data should be included to report the data underlying figures according to the guidelines set out in the authorship guidelines on Data Presentation.

2. Captions
Each figure caption should contain the following information, for each panel where they are relevant:
- a specification of the experimental system investigated (eg cell line, species name).
- the assay(s) and method(s) used to carry out the reported observations and measurements.
- an explicit mention of the biological and chemical entity(ies) that are being measured.
- an explicit mention of the biological and chemical entity(ies) that are altered/varied/perturbed in a controlled manner.
- the exact sample size (n) for each experimental group/condition, given as a number, not a range;
- a description of the sample collection allowing the reader to understand whether the samples represent technical or biological replicates (including how many animals, litters, cultures, etc.).
- a statement of how many times the experiment shown was independently replicated in the laboratory.
- definitions of statistical methods and measures:
  - common tests, such as t-test (please specify whether paired vs. unpaired), simple χ2 tests, Wilcoxon and Mann-Whitney tests, can be unambiguously identified by name only, but more complex techniques should be described in the methods section;
  - are tests one-sided or two-sided?
  - are there adjustments for multiple comparisons?
  - exact statistical test results, e.g., P values = x but not P values < x;
  - definition of 'center values' as median or average;
  - definition of error bars as s.d. or s.e.m.

Please complete ALL of the questions below.
Select "Not Applicable" only when the requested information is not relevant for your study.

Materials

Newly Created Materials	Information included in the manuscript?	In which section is the information available? (Reagents and Tools Table, Materials and Methods, Figures, Data Availability Section)
New materials and reagents need to be available; do any restrictions apply?	Not Applicable	

Antibodies	Information included in the manuscript?	In which section is the information available? (Reagents and Tools Table, Materials and Methods, Figures, Data Availability Section)
For **antibodies** provide the following information:<br>- Commercial antibodies: RRID (if possible) or supplier name, catalogue number and or/clone number<br>- Non-commercial: RRID or citation	Yes	Materials and Methods

DNA and RNA sequences	Information included in the manuscript?	In which section is the information available? (Reagents and Tools Table, Materials and Methods, Figures, Data Availability Section)
**Short novel DNA or RNA including primers, probes**: provide the sequences.	Yes	Materials and Methods, Appendix Table S1

Cell materials	Information included in the manuscript?	In which section is the information available? (Reagents and Tools Table, Materials and Methods, Figures, Data Availability Section)
**Cell lines:** Provide species information, strain. Provide accession number in repository **OR** supplier name, catalog number, clone number, and/**OR** RRID.	Yes	Materials and Methods
**Primary cultures:** Provide species, strain, sex of origin, genetic modification status.	Not Applicable	
Report if the cell lines were recently **authenticated** (e.g., by STR profiling) and tested for mycoplasma contamination.	Yes	tested in house in a routing basis

Experimental animals	Information included in the manuscript?	In which section is the information available? (Reagents and Tools Table, Materials and Methods, Figures, Data Availability Section)
**Laboratory animals or Model organisms:** Provide species, strain, sex, age, genetic modification status. Provide accession number in repository **OR** supplier name, catalog number, clone number, **OR** RRID.	Not Applicable	
**Animal observed in or captured from the field:** Provide species, sex, and age where possible.	Not Applicable	
Please detail **housing and husbandry conditions**.	Not Applicable	

Plants and microbes	Information included in the manuscript?	In which section is the information available? (Reagents and Tools Table, Materials and Methods, Figures, Data Availability Section)
**Plants:** provide species and strain, ecotype and cultivar where relevant, unique accession number if available, and source (including location for collected wild specimens).	Not Applicable	
**Microbes:** provide species and strain, unique accession number if available, and source.	Not Applicable	

Human research participants	Information included in the manuscript?	In which section is the information available? (Reagents and Tools Table, Materials and Methods, Figures, Data Availability Section)
If collected and within the bounds of privacy constraints report on age, sex and gender or ethnicity for all study participants.	Not Applicable	

Core facilities	Information included in the manuscript?	In which section is the information available? (Reagents and Tools Table, Materials and Methods, Figures, Data Availability Section)
If your work benefited from core facilities, was their service mentioned in the acknowledgments section?	Yes	Acknowledgements

Design

Study protocol	Information included in the manuscript?	In which section is the information available? (Reagents and Tools Table, Materials and Methods, Figures, Data Availability Section)
If study protocol has been **pre-registered, provide DOI in the manuscript**. For clinical trials, provide the trial registration number **OR** cite DOI.	Not Applicable	
Report the **clinical trial registration number** (at ClinicalTrials.gov or equivalent), where applicable.	Not Applicable	

Laboratory protocol	Information included in the manuscript?	In which section is the information available? (Reagents and Tools Table, Materials and Methods, Figures, Data Availability Section)
Provide DOI OR other citation details if **external detailed step-by-step protocols** are available.	Not Applicable	

Experimental study design and statistics	Information included in the manuscript?	In which section is the information available? (Reagents and Tools Table, Materials and Methods, Figures, Data Availability Section)
Include a statement about **sample size** estimate even if no statistical methods were used.	Not Applicable	
Were any steps taken to minimize the effects of subjective bias when allocating animals/samples to treatment (e.g. **randomization procedure**)? If yes, have they been described?	Not Applicable	
Include a statement about **blinding** even if no blinding was done.	Not Applicable	
Describe **inclusion/exclusion criteria** if samples or animals were excluded from the analysis. Were the criteria pre-established?<br><br>If sample or data points were omitted from analysis, report if this was due to attrition or intentional exclusion and provide justification.	Not Applicable	
For every figure, are **statistical tests** justified as appropriate? Do the data meet the assumptions of the tests (e.g., normal distribution)? Describe any methods used to assess it. Is there an estimate of variation within each group of data? Is the variance similar between the groups that are being statistically compared?	Yes	Materials and Methods, Figure legends

Sample definition and in-laboratory replication	Information included in the manuscript?	In which section is the information available? (Reagents and Tools Table, Materials and Methods, Figures, Data Availability Section)
In the figure legends: state number of times the experiment was **replicated** in laboratory.	Yes	
In the figure legends: define whether data describe **technical or biological replicates**.	Yes	

Ethics

Ethics	Information included in the manuscript?	In which section is the information available? (Reagents and Tools Table, Materials and Methods, Figures, Data Availability Section)
Studies involving **human participants**: State details of **authority granting ethics approval** (IRB or equivalent committee(s), provide reference number for approval.	Not Applicable	
Studies involving **human participants**: Include a statement confirming that **informed consent** was obtained from all subjects and that the experiments conformed to the principles set out in the WMA Declaration of Helsinki and the Department of Health and Human Services Belmont Report.	Not Applicable	
Studies involving **human participants:** For publication of **patient photos**, include a statement confirming that consent to publish was obtained.	Not Applicable	
Studies involving experimental **animals**: State details of **authority granting ethics approva**l (IRB or equivalent committee(s), provide reference number for approval. Include a statement of compliance with ethical regulations.	Not Applicable	
Studies involving **specimen and field samples:** State if relevant **permits** obtained, provide details of authority approving study; if none were required, explain why.	Not Applicable	

Dual Use Research of Concern (DURC)	Information included in the manuscript?	In which section is the information available? (Reagents and Tools Table, Materials and Methods, Figures, Data Availability Section)
Could your study fall under dual use research restrictions? Please check biosecurity documents and list of **select agents and toxins** (CDC): https://www.selectagents.gov/sat/list.htm	Not Applicable	
If you used a select agent, is the security level of the lab appropriate and reported in the manuscript?	Not Applicable	
If a study is subject to dual use research of concern regulations, is the name of the **authority granting approval and reference number** for the regulatory approval provided in the manuscript?	Not Applicable	

Reporting

The MDAR framework recommends adoption of discipline-specific guidelines, established and endorsed through community initiatives. Journals have their own policy about requiring specific guidelines and recommendations to complement MDAR.

Adherence to community standards	Information included in the manuscript?	In which section is the information available? (Reagents and Tools Table, Materials and Methods, Figures, Data Availability Section)
State if relevant guidelines or checklists (e.g., **ICMJE, MIBBI, ARRIVE, PRISMA**) have been followed or provided.	Not Applicable	
For **tumor marker prognostic studies,** we recommend that you follow the **REMARK** reporting guidelines (see link list at top right). See author guidelines, under 'Reporting Guidelines'. Please confirm you have followed these guidelines.	Not Applicable	
For **phase II and III randomized controlled trials**, please refer to the **CONSORT** flow diagram (see link list at top right) and submit the CONSORT checklist (see link list at top right) with your submission. See author guidelines, under 'Reporting Guidelines'. Please confirm you have submitted this list.	Not Applicable	

Data Availability

Data availability	Information included in the manuscript?	In which section is the information available? (Reagents and Tools Table, Materials and Methods, Figures, Data Availability Section)
Have **primary datasets** been deposited according to the journal's guidelines (see 'Data Deposition' section) and the respective accession numbers provided in the Data Availability Section?	Yes	Data Availability Section
Were **human clinical and genomic datasets** deposited in a public access-controlled repository in accordance to ethical obligations to the patients and to the applicable consent agreement?	Not Applicable	
Are **computational models** that are central and integral to a study available without restrictions in a machine-readable form? Were the relevant accession numbers or links provided?	Not Applicable	
If publicly available data were reused, provide the respective **data citations in the reference list**.	Not Applicable	
